# Hippocampal CA1 Ripples as Inhibitory Transients

**DOI:** 10.1371/journal.pcbi.1004880

**Published:** 2016-04-19

**Authors:** Paola Malerba, Giri P Krishnan, Jean-Marc Fellous, Maxim Bazhenov

**Affiliations:** 1 Department of Cell Biology and Neuroscience, University of California Riverside, Riverside, California, United States of America; 2 Department of Psychology, University of Arizona, Tucson, Arizona, United States of America; The Krasnow Institute for Advanced Studies, UNITED STATES

## Abstract

Memories are stored and consolidated as a result of a dialogue between the hippocampus and cortex during sleep. Neurons active during behavior reactivate in both structures during sleep, in conjunction with characteristic brain oscillations that may form the neural substrate of memory consolidation. In the hippocampus, replay occurs within sharp wave-ripples: short bouts of high-frequency activity in area CA1 caused by excitatory activation from area CA3. In this work, we develop a computational model of ripple generation, motivated by *in vivo* rat data showing that ripples have a broad frequency distribution, exponential inter-arrival times and yet highly non-variable durations. Our study predicts that ripples are not persistent oscillations but result from a transient network behavior, induced by input from CA3, in which the high frequency synchronous firing of perisomatic interneurons does not depend on the time scale of synaptic inhibition. We found that noise-induced loss of synchrony among CA1 interneurons dynamically constrains individual ripple duration. Our study proposes a novel mechanism of hippocampal ripple generation consistent with a broad range of experimental data, and highlights the role of noise in regulating the duration of input-driven oscillatory spiking in an inhibitory network.

## Author Summary

Our memories are consolidated while we sleep through a bidirectional exchange of information between two brain areas called cortex and hippocampus. Neurons that were active in behavioral tasks reactivate again in both structures during sleep in a process of linking and modifying memories from the short term storage of the hippocampus to permanent storage in the neocortex. This process occurs mainly during short oscillatory hippocampal electrical events called sharp wave-ripples. We propose a novel mechanism of ripple generation consistent with a wide range of experimental data, to explain how hippocampal network properties shape ripple frequency and duration. Understanding the neuronal mechanism underlying ripples is crucial to explaining how the interaction between hippocampus and cortex during sleep enables memory consolidation.

## Introduction

Sleep, which consumes about a third of our lives, is thought to play a critical role in memory consolidation. Specifically, sleep influences unconscious post-encoding processes that result in long term memory consolidation and reconsolidation. Behavioral studies show that performance in various memory tasks improves after sleep compared to a similar period of wake [1, 2], and such improvement was observed in declarative, procedural and emotional memory tasks [3–7].

Cortical and hippocampal circuits show characteristic oscillatory activities at different sleep stages [1]. During slow-wave sleep (SWS), cortex is synchronized by low-frequency slow oscillations (0.2–1 Hz) between Down states–in which most cells are hyperpolarized–and Up states, in which firing activity is intense and cells are depolarized [8]. The hippocampus generates sharp wave-ripple complexes (SWR), in which a strong excitatory input from CA3 pyramidal cells leads to broadly distributed postsynaptic potentials (the sharp waves) in CA1 stratum radiatum, while the pyramidal layer shows a quick bout of high frequency LFP activity (the ripple) [9–11]. Ripples exist both in a quiet awake state and during slow-wave sleep, and disruption of ripple activity is known to impair memory [12, 13].

In the rat, one of the mechanisms thought to underlie memory consolidation is place cell replay: a phenomenon in which the pattern of relative firing of hippocampal pyramidal cells that code for position (place cells) re-occurs during post-task sleep [14, 15]. Importantly, hippocampal replay has been shown to take place during CA1 ripples, on very short time scales during which synaptic plasticity is likely to arise. Interestingly, SWRs are more likely to occur during cortical Up states [16] and may potentially influence the spatio-temporal pattern of Up state generation. Thus, understanding the process of ripple generation is a crucial step towards identifying the mechanism of brain-wide sleep-dependent memory consolidation.

In this work, we propose a novel mechanism of CA1 ripple generation during sleep. Our *in vivo* data show that ripples have a broad frequency distribution, exponential inter-arrival times and a highly non-variable duration. In our model, high-frequency firing in perisomatic interneurons is caused by input from area CA3, and mediates high-frequency local field potential (LFP) oscillations in CA1 pyramidal neurons. The main novelty of this model, compared to ones already proposed in the literature [17–19] (see [20] for a review), is the prediction of the ability of CA1 to self-time ripple durations, and hence limit the extent of replay in a dynamic fashion, from ripple to ripple. We propose that phase-dispersion (loss of synchrony) induced by noise on the oscillatory dynamics constrains the duration of a ripple event. This minimal model is not only able to explain experimental data regarding basic ripple properties, but is also consistent with recent data on ripples and ripple-like activity triggered by optogenetic stimulations *in vivo* [21].

This paper is organized as follows: we first introduce the experimental results, then describe our computational model and show that it can produce ripple-like oscillations. We then use those observations to inform predictions that can be made by the full model. Next we study the role of interneurons in setting inhibitory transient mechanisms underlying ripple oscillations in the model, and the role of pyramidal cells in the overall ripple structure. We conclude showing that selective input from CA3 can induce sequential reactivation of CA1 pyramidal cells during ripples in our model.

## Results

### Data description: Ripples duration in CA1 has narrow variance

Data shows that ripples are local events [22], and that a given CA1 pyramidal cell rarely spikes more than once per ripple oscillation [10, 23, 24]. Many pyramidal cells are not recruited by ripples recorded across different sessions, while some are recruited by almost all events. On average 10% of pyramidal cells are active in any given ripple, and their spikes are locked to the trough of each the oscillations within a ripple event [25, 26]. On the other hand, inhibitory interneurons in the pyramidal layer spike across the ripple, with a firing rate consistent with the ripple frequency [23, 24]. This suggests a predominant role for pyramidal layer interneurons in organizing CA1 ripple firing. Moreover, spiking in CA3 is not locked to CA1 ripples [27, 28], which further advances the idea that ripples are an intrinsic CA1 rhythm, which can be initiated by sufficiently strong incoming inputs.

**Fig 1:**
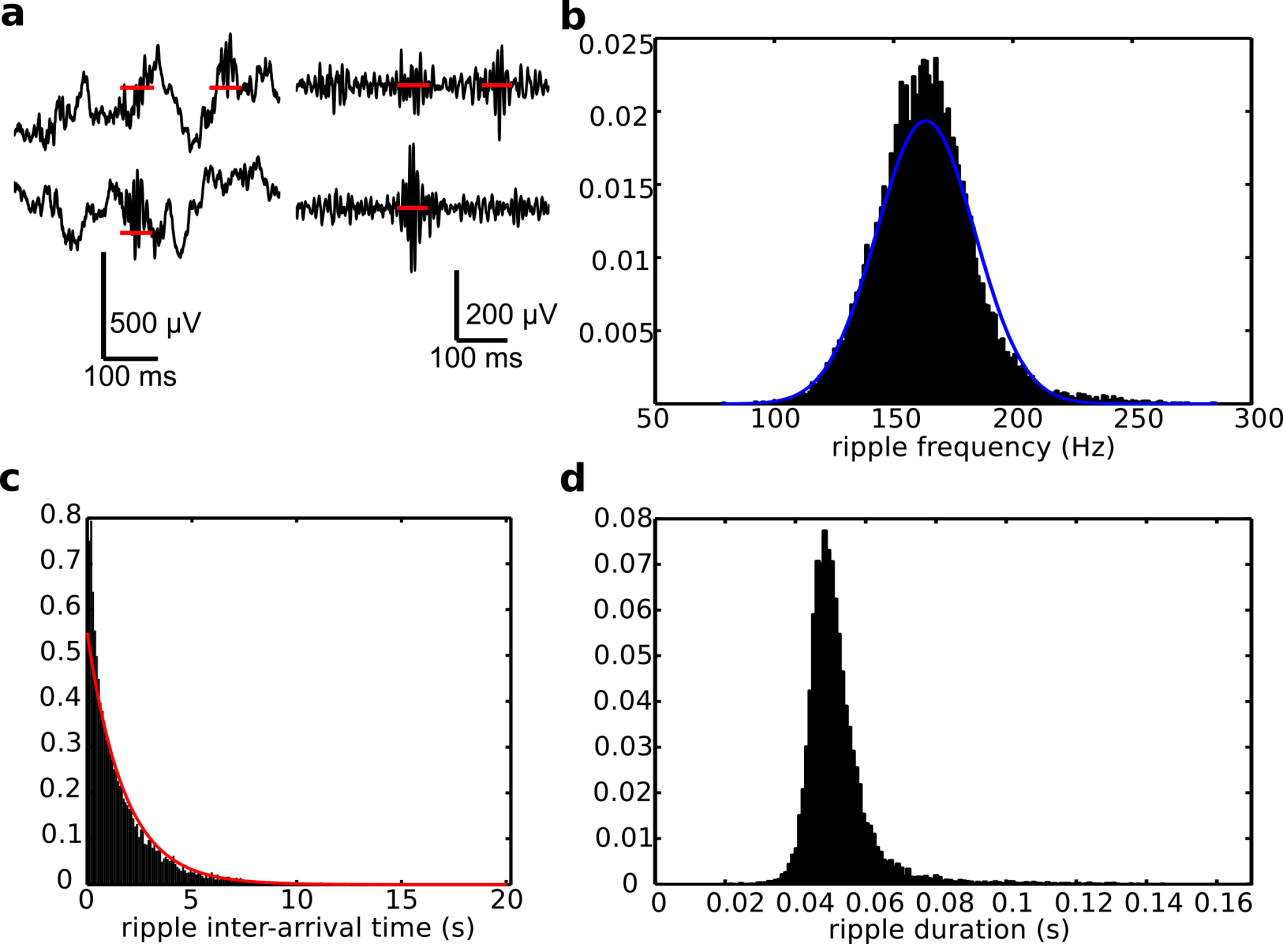
CA1 ripples have standardized durations. From a 25hr experiment recording, ripples are identified as large amplitude excursion of the band-passed LFP. (a) The leftmost plots show wide band (1-400Hz) LFP recordings from CA1. The rightmost plots show the same LFPs band-pass filtered between 50Hz and 350Hz. (b) Histogram of ripple frequencies (count normalized to total number of ripples). The blue line is its Gaussian distribution fit. (c) Histogram of ripple inter-arrival times, and exponential fit in red. (d) Histogram of ripple durations, showing a high kurtosis, hence highlighting that ripples are very different in frequency and have almost memory-less arrival times, but their duration has small variability.

We started investigating the nature of ripple oscillations by studying LFP recordings in hippocampal CA1 in rats. Representative examples of the wide-band and band-passed recordings are shown in Fig 1A. We focused on a few salient characteristics of ripple waves, such as frequency, duration and inter-arrival time (defined as the time between a ripple event and the next). Fig 1 shows that ripple frequency, defined as the inverse of the average inter-peak interval during a ripple event, was normally distributed around 163.5 (± 20.6) Hz (Fig 1B), their inter-arrival times were approximately exponential, with fitted rate 1.7748 Hz (Fig 1C), and their duration (Fig 1C) was centered about 51 (± 9.4) ms showing a high-kurtosis distribution (K = 20.1952, where for a normal distribution K = 3) [28]. Note that ripple frequency is representative of the peak-to-peak time within a given ripple (see S1 Fig for a representation), while the count of ripple events in a given time interval would be called ripple density, and can be found as the inverse of the inter-arrival times in such interval.

Ripples are events specific to the pyramidal layer of CA1, and ripples simultaneously recorded across different tetrodes appear to have amplitudes that vary independently [22]. This suggests that ripples are local events within the CA1 pyramidal layer [22]. Furthermore, data show that ripple events turn to epileptic activity when GABA_A_ is blocked in CA1 slices [29] suggesting that interneurons limit the extent and sculpt the frequency content of these events. The population of pyramidal cells is most active close to the peak of a ripple event, which is defined as the time when the filtered LFP reaches its maximum amplitude (often the biggest trough). Moreover, only a few pyramidal cells are recruited by ripples in CA1. On the other hand, most basket cell interneurons spike in ripples, and across the event duration [23, 24].

Our data revealed that the length of time between successive ripples is not dependent on any feature of the current ripple (frequency or duration). In fact, scatter plots of ripple frequency vs time-to-next ripple, and ripple duration vs time-to-next ripple, did not show any particular correlation (see S2 Fig in supporting information). Poincaré return maps show that both ripple frequency and duration are not dependent on the frequency or duration of the preceding ripples (S2 Fig). This implies that we can model each ripple in CA1 as an event directly triggered by an incoming CA3 input volley. Hence, we looked for a ripple mechanism that can generate Gaussian-distributed frequencies for a fairly constrained time interval.

### Computational model of CA1 ripples induced by CA3 input

**Fig 2:**
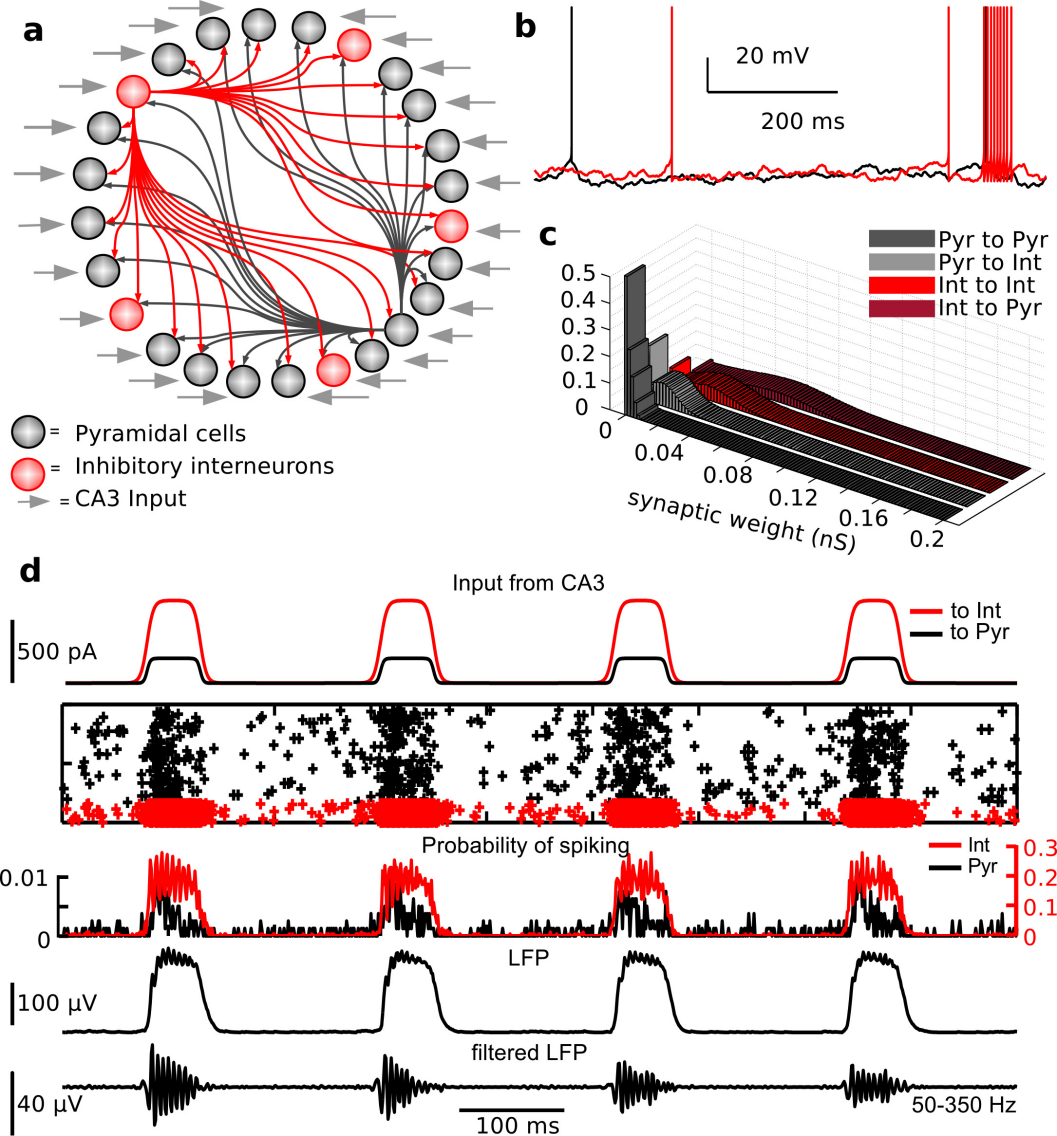
Computational model of CA1 ripples. (a) Model schematic. Parameters: see Materials and Methods. (b) Example voltage traces of pyramidal cell (black) and interneuron (red). (c) Distributions of synaptic weights. (d) A simulation, described from the top. Traces of current inputs from CA3 to pyramidal cells (black) and to inhibitory interneurons (red). Rastergram where cells are stacked along the y-axis and crosses represent spikes of pyramidal cells (black) and interneurons (red). Probability to spike for pyramidal cells (black) and interneurons (red) in 1ms bins. LFP wide-band, and band-passed filtered (50-350Hz).

We reasoned that our model needed to represent a small patch of CA1, reached by strong incoming excitation from CA3, so that the activity of all cells we modeled would be picked up by a single electrode. Given that ripples are measured in the pyramidal layer, we chose to model only pyramidal cells and parvalbumin positive basket cells. In fact, other interneuron types that might be active during sharp waves impinge on pyramidal cells at different layers [30, 31], which do not show ripple frequency oscillations. Hence, they might have a modulatory influence on the overall ripple appearance but are not in a position to set the pace of ripple frequency. The network consisted of 800 pyramidal cells and 160 interneurons, a ratio in agreement with CA1 anatomy [32], and we used all-to-all connectivity, with the exception of the synapses between pyramidal cells, which were few and much weaker than all others [33], consistent with CA1 anatomy [34]. Fig 2 shows a network representation (Fig 2A), example traces from a pyramidal cell and an interneuron (Fig 2B), and the distributions of synaptic weights (Fig 2C) in the model.

To model each neuron, we used the Adaptive Exponential Integrate-and-fire model [35], as it is simple (only two variables) and has been shown to reproduce many different spiking behaviors [36], because of the expressed essential non-linearities [37]. To account for heterogeneity, each neuron received a different independent Ornstein-Uhlenbeck (OU) noise and a mean DC current to set baseline excitability (see Materials and Methods for details). The noise term represents the *in vivo* state of the voltage in each cell, which is likely receiving a much higher barrage of synaptic inputs than the one provided by the network spiking activity in our model. The OU process, which can be thought of as a filtered white noise process, is used in dynamic-clamp experiments to mimic *in vivo* state in hippocampal slice recordings [38]. We assigned fast time scales to the synapses, in agreement with recent *in vitro* estimates [39]. The average synaptic strengths values were chosen to induce post-synaptic potentials of less than 1mV. To represent the integrated input from CA3 localized in time, we delivered input current to all cells, with different magnitudes for pyramidal cells and interneurons, due to the lower input resistance of pyramidal cells [40, 41] (see the first panel of Fig 2D; details in Materials and Methods). In the text below we will refer to this current as CA3 input to the CA1 network. All details of model implementation and justifications for specific parameter choices are reported in Materials and Methods.

The rastergram and spike probability curves in Fig 2D show that when inputs from CA3 reached a patch of CA1, high-frequency firing was triggered in the interneuron population, which self-organized in oscillations. Firing in pyramidal cells increased as well, but the probability of firing for the pyramidal cell population was much smaller than for interneurons (0.2 vs 0.005%). To compare our model to experimentally recorded ripples, we approximated the LFP in the pyramidal layer using the average net synaptic input (from both excitatory and inhibitory cells) received by all pyramidal cells, and derived a wideband LFP signal (Fig 2D). The model generated ripple-like oscillations that could be detected by band-passing our LFP estimate, shown in the bottom panel of Fig 2D.

**Fig 3:**
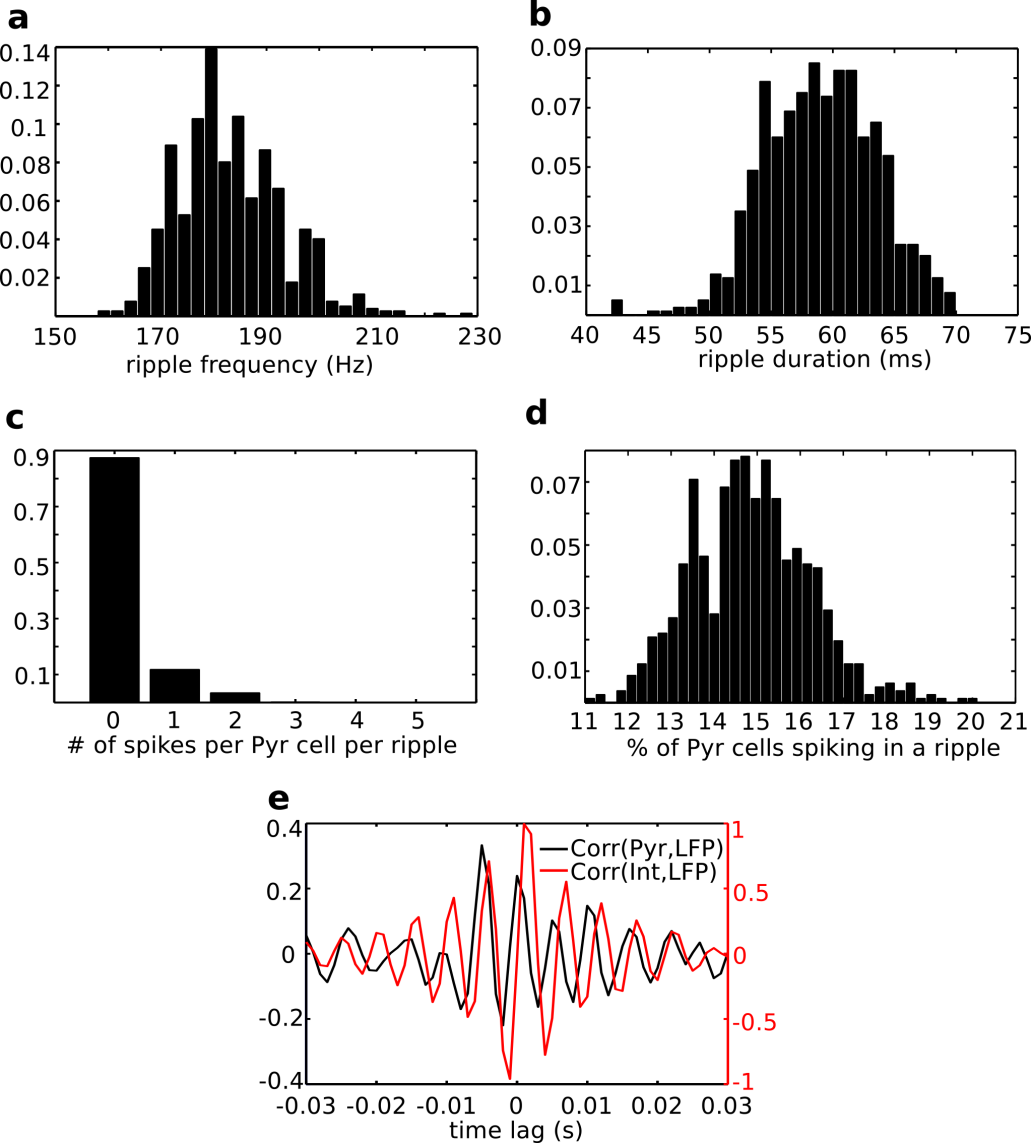
Properties of ripples in the model. Histograms of ripple frequency (a), durations (b), number of spikes of each pyramidal cell during ripples (c) and percent of pyramidal cells spiking in a ripple (d). (e) Average cross-correlations between firing probability of interneurons (red) or pyramidal cells (black) and the band-passed LFP.

Ripples produced by our computational model have properties consistent with the ones recorded *in vivo*. Fig 3 shows that the mean frequency for a given input intensity is 162.4 ± 12.5 Hz (Fig 3A), and ripple duration is 57.2 ± 3.1 ms (Fig 3B). Also, most pyramidal cells do not spike during a ripple, in fact on average a ripple shows spikes from 14.76% of the pyramidal cell population (Fig 3D), and those that do will not spike across the ripple duration, but only once (Fig 3C) in agreement with previous experimental observations [42]. Furthermore, the spiking activity of pyramidal cells is known to precede the spiking of interneurons within each ripple wave [43]. We tested this property in our model by computing the cross-correlation between the filtered LFP and the firing probability of each cell population, averaged across 40 ripples. Fig 3E shows that peaks in the correlation between pyramidal cell spiking and LFP preceded those for interneuron activity across ripple waves. For completeness, we also verified that there was no inherent rhythmic activity in the network background state that could be inducing this relationship within ripples beyond the mechanistic phenomena we report (S3 Fig). This model is consistent with CA1 activity during non-REM sleep *in vivo*, when ongoing theta-gamma activity is not present in the background.

### Model predictions: CA1 as a local processing unit, refining synchronous CA3 output

We next studied the main properties of the dynamics of ripple oscillations in our model, looking for intrinsic CA1 properties that played a role in shaping ripple spiking. We found that CA1 properties determine ripple duration, while ripple frequency is not controlled by the time scale of inhibitory synapses. Also, the amount of pyramidal cells spiking during ripples is determined by competing forces: the excitatory drive they receive from CA3 and the amount of local inhibition they receive from the CA1 inhibitory population.

**Fig 4:**
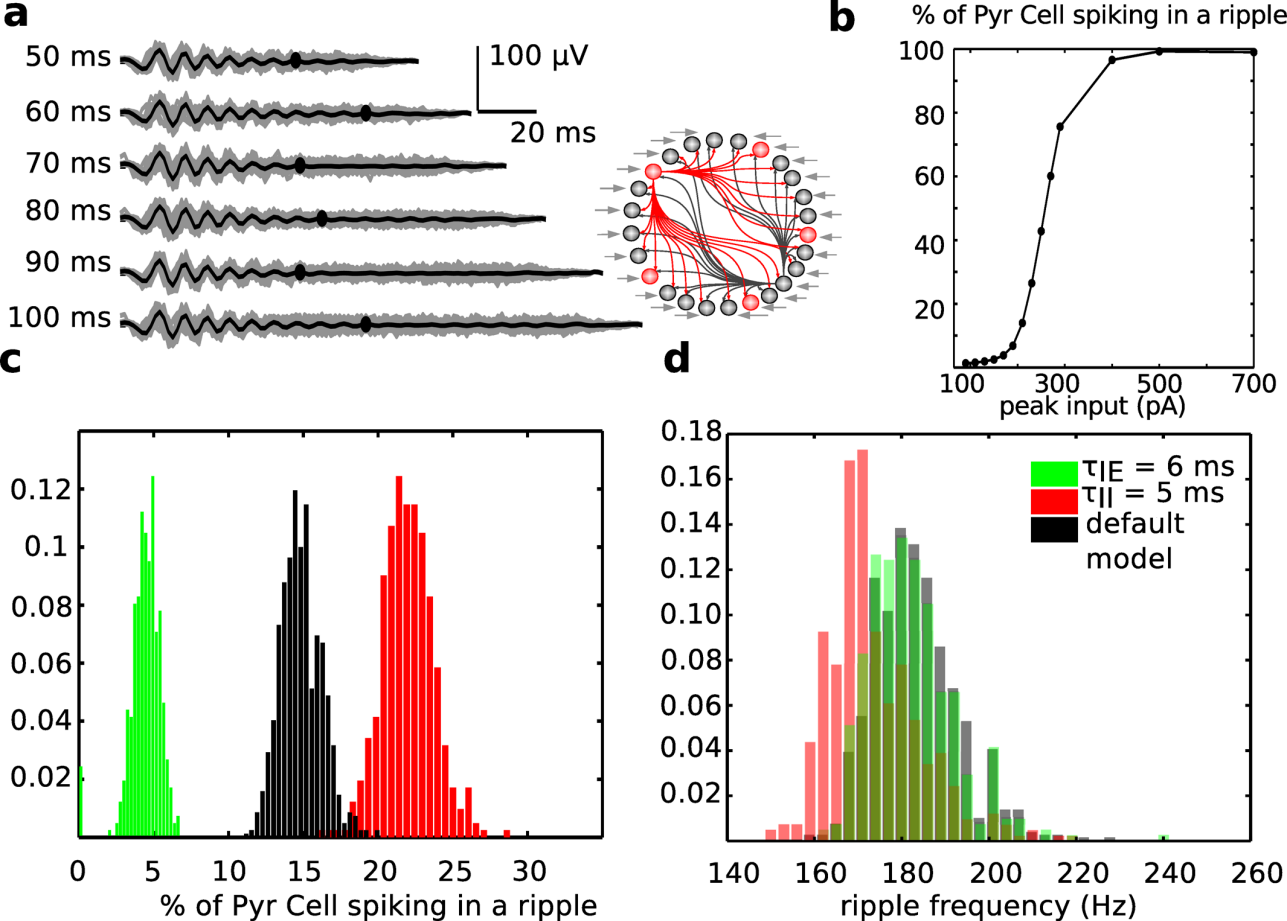
Predictions from ripple network model. (a) Stereotypical ripple LFP has a duration independent from CA3 input length. On the left, the duration of CA3 input is reported. In each group, shown in gray are the actual band-passed LFPs for 40 ripples, while the black line is their average. The black dot marks where each average ripple ends. Next to the LFPs, a schematic of the full network used in this figure (as in Fig 2) (b). The percentage of pyramidal cells spiking on every ripple increases with the magnitude of CA3 input to pyramidal cells population. (c) Effect of inhibition on pyramidal cell recruitment to ripples. Histograms of the percent of pyramidal cells spiking in any given ripple in three conditions: default (black bars), inhibition on interneurons increased to 5ms (red bars), inhibition on pyramidal cells increased to 6ms (green bars). Bars heights have been normalized by total number of ripples. Note that increasing inhibition on interneurons un-inhibits pyramidal cell spiking, while increased inhibition on pyramidal cells predictably suppresses pyramidal cell firing during ripples. (d) Increasing inhibition time scale has almost no effect on ripple frequency. Histograms of ripple frequency under the same three conditions as in panel c.

Since the input current (representing the sum of synchronized spiking in CA3) caused ripples to initiate, we asked if ripples would continue oscillating for as long as the input was present. Fig 4A shows that the ripple LFP duration stayed un-varied independently of the different CA3 input durations we tested. The band-passed LFPs for 40 ripples across different input durations are shown in gray, while the black line is their average. The graph shows that even if spiking was still enhanced for the duration of CA3 inputs, the organized oscillatory activity was lost after about 60 ms for all cases considered. This emphasizes that CA1 can control ripple duration, even if it cannot control their initiation.

Pyramidal cell spiking within a ripple is responsible for carrying information to downstream areas. Hence, it is important to understand what factors regulate the overall recruitment of CA1 pyramidal cells to a given ripple in our model. The ratio of CA1 pyramidal cells recruited in a given ripple in our model is modulated by both excitatory drive from CA3 on this population and inhibitory currents within CA1 [44]. In fact, increasing CA3 input to this population raised the percentage of pyramidal cells spiking on every ripple (Fig 4B, doubling the peak value takes the recruitment percentage from 14% to above 90%), and increasing the time scale of inhibition onto pyramidal cells reduced their recruitment (Fig 4C, setting τ = 6ms results in an average 4.4% of pyramidal cells spiking in any given ripple). Fig 4C also shows that increasing inhibition onto interneurons resulted in higher pyramidal cell excitability, because the inhibitory population activity was reduced overall. Thus, a net change in the GABA_A_ time scale has competing effects on pyramidal cells recruitment to ripple activity, leaving the fine regulatory function to highly selective CA3 input. The predominance of CA3 input over local inhibition in choosing which pyramidal cells are recruited to a specific ripple is consistent with the known synaptic plasticity at Shaffer collateral synapses between CA3 and CA1 pyramidal cells [45]. Furthermore, we found that changing the time scale of inhibition did not drastically slow ripple frequency (Fig 4D).

### Role of inhibitory neurons: CA1 ripples mechanism

To better understand mechanism underlying the dynamics of ripple oscillations, we next moved to studying a simplified system. Since pyramidal cells typically spike less and at much lower frequencies than inhibitory cells during ripples, we started from studying the role of interneurons in the ripple dynamics we observed in the full model. To do that, we considered a network of only inhibitory neurons receiving a step of DC current, and studied the resulting input-driven high-frequency population firing.

**Fig 5:**
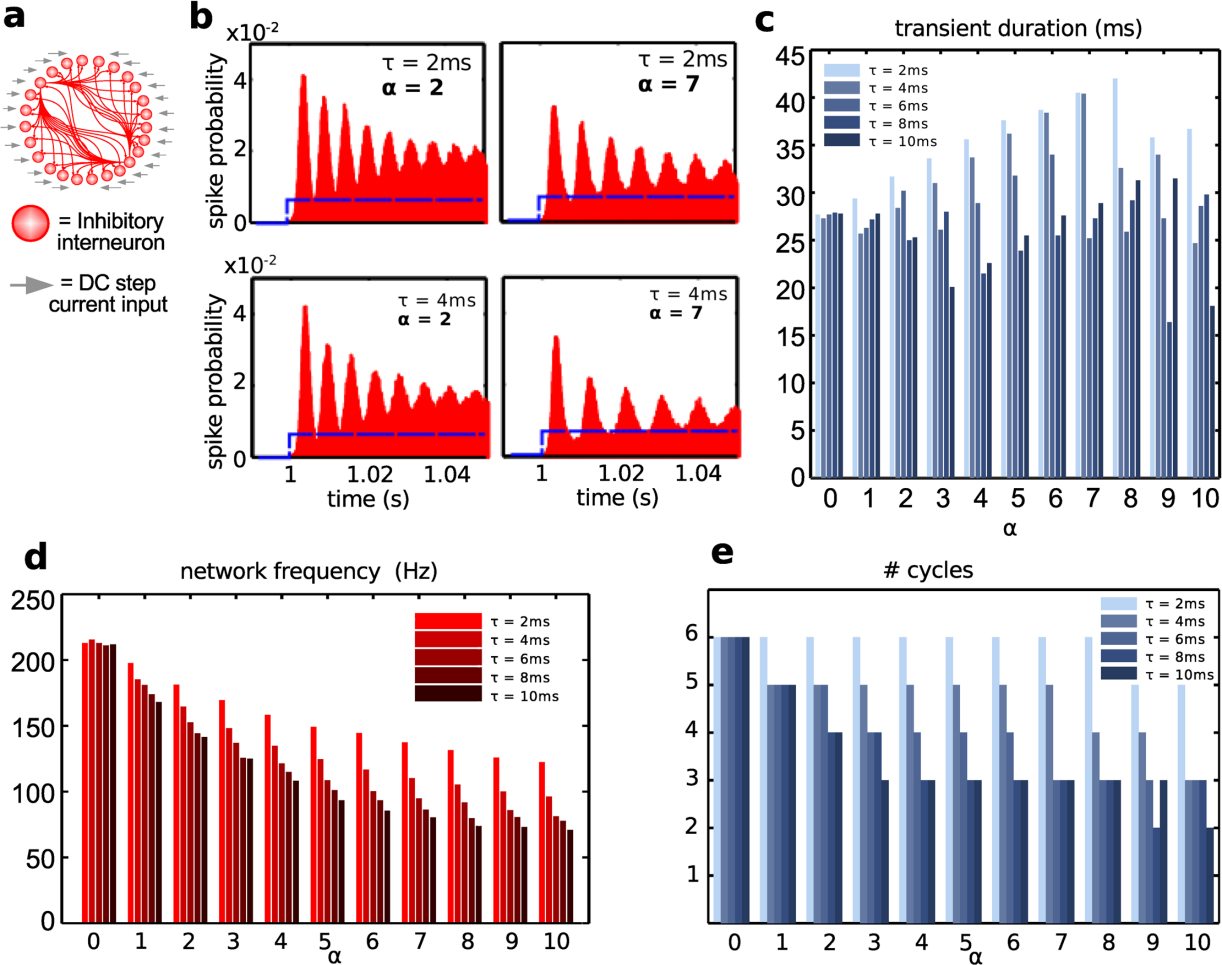
Transients in inhibitory network explain ripple mechanism. (a) Schematic representation of the model considered, only composed of inhibitory neurons. (b) Histograms of spike probabilities, with step current input (blue). The time axes is binned in 1ms bins. Changing parameters: decay time scale of inhibitory synapses (τ) and scaling (α) of maximal inhibitory synapses conductance. (c) Transient duration (in ms) as a function of α and τ. (d) Transient frequency. (e) Number of peak cycles in the transient, before the distribution flattens (see Materials and Methods).

The step of DC current delivered to all inhibitory interneurons amounted to the same value as the peak input current from CA3 to interneurons in the full model (700 pA). To construct the profile of spiking probability in response to the current step, we run 100 simulations for each parameter set, and built the cumulative histogram of probability of spiking as a function of time. Fig 5A shows a schematic of the reduced network, while Fig 5B shows that the common input step (at time = 1s) initially organized the network as indicated by the rhythmic oscillations of the population activity. The amplitude of these oscillations progressively decreased, indicating a transient nature of the high-frequency activity, which was de-synchronized by the intrinsic noise. Eventually, the population firing rate stopped oscillating and settled to a mean constant value, which depended on the size of the current step. This behavior of the isolated interneuron population is consistent with data [46]: *in vitro* optogenetic experiments (albeit in area CA3) show that activating only parvalbumin positive interneurons with a step of light, of duration up to 50ms, results in an average oscillatory behavior in which the peaks are progressively attenuated in time.

Once we found that synchronous inputs organize transient oscillatory patterns in our purely inhibitory network, we asked what role synaptic inhibition could play in setting the properties of this transient oscillation. We reasoned that the role of inhibitory synapses in network behavior could be twofold: they promote synchrony while enough synaptic inputs are aligned (early after the initiation of the step of current), but synaptic currents switch to propagating de-synchronization if enough neurons are asynchronous. We numerically studied the role of inhibition in transient high-frequency synchronization by changing two main parameters: the decay time scale of the inhibitory synaptic conductance (τ) and the strength of synaptic inhibition. To address synaptic strengths while respecting the choice of a normal distribution of synaptic conductances around a mean, we introduced a non-dimensional scaling factor α, which we systematically varied. When α = 0, no synapse was active, and when α = 2 all synapses were twice as strong as their baseline values. Fig 5B is provided to show the effect of changing the inhibitory signal: the overall impact of inhibition on the oscillation pattern depended on the decay time scale τ, or synaptic strength α, or both. As one can see in Fig 5, changing strength of synaptic inhibition in the inhibitory network affects both the duration of transient synchronization (Fig 5C) (defined as a time window when amplitude of oscillations is still high, see Materials and Methods, Analysis of inhibitory network model) and its frequency (Fig 5D) (defined as the inverse of the average peak-to-peak time delay, within transient duration). Because of that, the number of oscillatory peaks within a transient changed as well (Fig 5E).

Fig 5 shows that frequency remained within ripple range for a time scale of inhibition within a broad range of 2–6 ms as long as synaptic weights were limited to within about 200% of the baseline value. Also, in the condition of α = 0, when the network is disconnected, one can still see the noise-induced de-synchronization; when the network has active and fast (small τ) synaptic connections the de-synchronization is delayed (Fig 5C). The increase of inhibition (through longer decay times or stronger conductances) resulted in the reduction of both the transient oscillation frequency (Fig 5D) and its peak count (Fig 5E). This is an important observation and it highlights the point that oscillations in this system are not a rhythm in the traditional sense of the word: they are not an oscillation that would persist in time as long as there are no changes to the CA1 model or the input, but rather a transient arising from a strong initial input capable of synchronizing CA1 neurons. The last point is critical: given that the stationary state of the network receiving a step current input is non-oscillatory, if the initial common step fails to synchronize enough neurons, then the transient oscillation would only last about 1 or 2 cycles, if at all.

**Fig 6:**
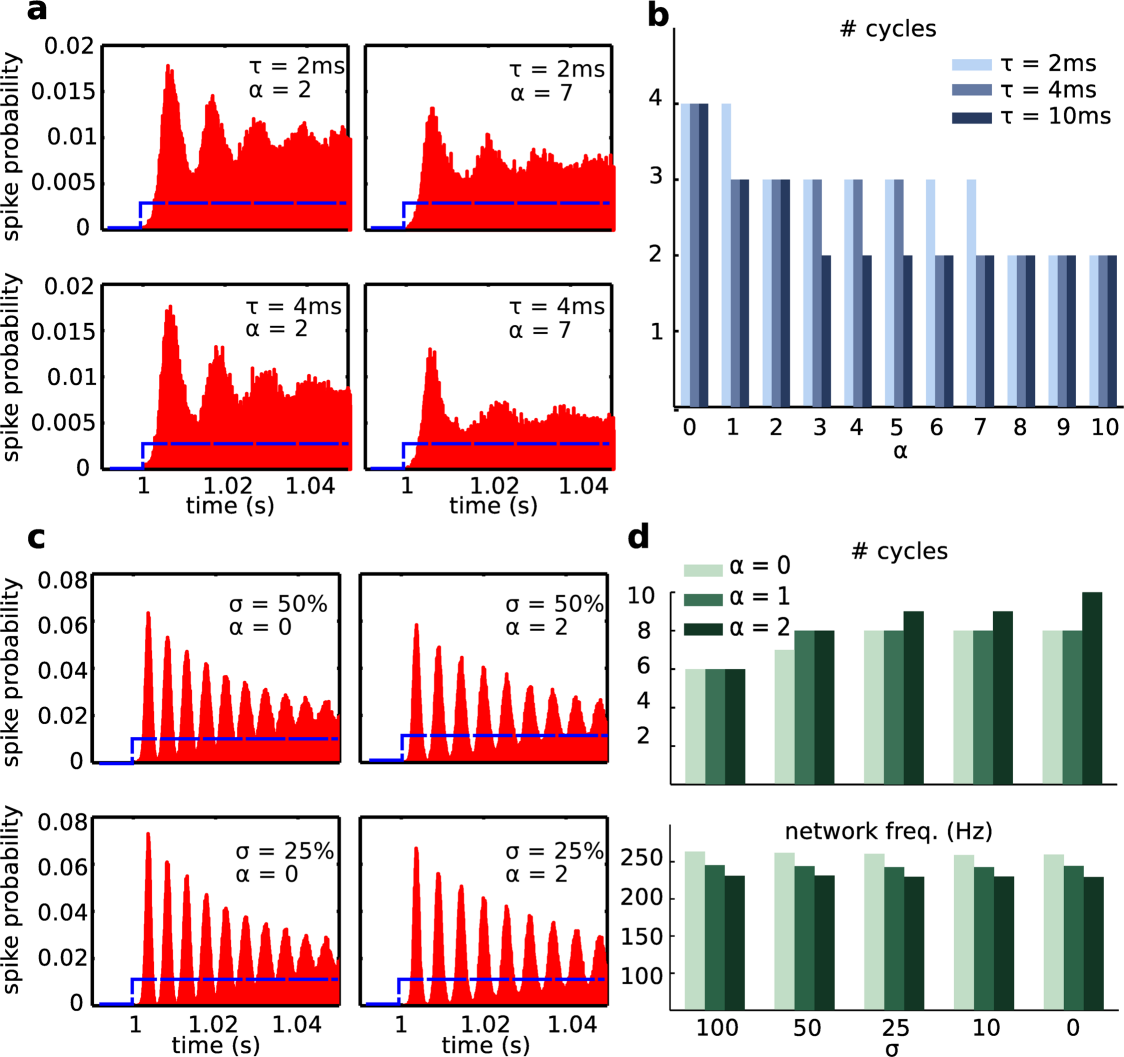
Transients in inhibitory network depend on input strength and noise size. (a, b) Effect of the input amplitude on oscillation properties. (a) Histograms of firing probabilities for a model shown in Fig 5, only with a DC step half the size (350 pA). The time axes is binned in 1ms bins. Note that synchrony is strongly affected, and the transient is composed by a drastically reduced number of cycles (b). (c, d) Effect of noise and strength of inhibition on transient properties. (c) Histograms of firing probabilities, for changing synaptic strength (scaled by α) and noise standard deviation (scaled by σ). The time axes is binned in 1ms bins. (d) Top panel: number of cycles composing the transient event. Bottom panel: network frequency.

To further explore this point, we studied how the transients organize when the initial step of the input current is halved (Fig 6A). In that case, much fewer interneurons were recruited to the initial synchronous population (note the scale of firing probability on the y-axes of Fig 6A). Inhibitory currents still affected oscillations, but the transient lasted very few cycles (Fig 6B) and peaks were smaller. We concluded that if the initial current step failed to synchronize a large enough population of neurons, the resulting slower oscillation faded in only 2 to 3 cycles. Hence, this network shows an all-or-none property: a smaller step of input that could in principle recruit lower frequency oscillations cannot recruit a transient at all. In fact, it takes an input of sufficient size to generate a transient that lasts enough cycles and recruits enough neurons for a fast oscillation to be visible in the LFP.

We next investigated the role of noise in setting the duration and frequency of oscillatory transients in the inhibitory network. We progressively reduced the noise coefficient using a scaling factor σ. Fig 6C shows the network behavior in the case of reduced noise amplitude, where spikes were far more synchronized around the network peaks compared to the 100% noise case shown in Fig 5B. Fig 6D shows that the duration of transient oscillations was controlled by the noise level (larger noise resulted in shorter transients), while frequency was not. In summary, the network oscillations we observed in our purely inhibitory network do not emerge out of interaction among the neurons, but rather from receiving a common, synchronizing, input. If the neurons were identical, received identical inputs, had no noise, and had no CA1 synapses then the oscillations would be trivially persistent, since all of the interneurons would remain in the phase locked state. The transient nature of this oscillation was due to network heterogeneity, introduced in our model via a different direct current to each neuron, and independent noise (where larger noise introduced more heterogeneity). Input strength, noise size, synaptic inhibition strength and duration are all factors that can control and tune the transient behavior reported in our model.

Our findings in the purely inhibitory network have direct implications for the full hippocampal model: due to the underlying transient mechanism that is highlighted by the dynamics of the reduced inhibitory network, local CA1 properties such as the level of noise and the strength of inhibitory synapses will control ripple duration, while the degree of synchrony of CA3 inputs (which is represented by the amplitude of the input current in our model) will determine whether a ripple occurs. In fact, if CA3 spikes were not synchronous enough, the net sum of the post-synaptic currents impinging on CA1 cells would be small, and hence the size of the CA3 current input we deliver to the model would be small as well. Therefore, the size of our current input from CA3 is effectively a model for synchrony in CA3 pyramidal cells spiking. Ripple frequency depends on balancing the number of interneurons recruited by the ongoing activity with the lateral inhibition known to exist between interneurons. Since, in principle, we could find oscillations in reduced model in the case of α = 0, where no synapses between the interneurons were present, we checked if removing I-to-I synapses in the full model would affect the properties of CA3-input driven ripples (S5 Fig). We found that ripple frequency transients can indeed be elicited in the network, however duration of ripples was generally reduced and the likelihood for occasional very short transients increased.

The inhibitory network also showed that an input step of current too small would result in very few (1 to 3) oscillatory peaks, effectively failing to induce the transient. In the full model, we verified that reduction of the input size (to both pyramidal cells and inhibitory neurons) resulted in decrease of the ripple amplitude and disruption of ripple events (S4 Fig). This was particularly evident when the input was scaled to 30% of its magnitude. As a result, the ripple, and consequently a bout of memory consolidation, only occurs if activity in CA3 is sufficiently synchronous, and results in a strong enough input to CA1. This ‘synchrony threshold’ ensures that uncorrelated CA3 inputs are essentially ignored by CA1. Furthermore, this means that a transient oscillation is triggered only at high frequencies above the gamma (30–90 Hz) range. As it is known that the interaction between pyramidal cells and basket cells in hippocampal CA1 region underlies gamma (30–90 Hz) oscillations [47, 48], the above findings suggests the mechanism recruiting ripple oscillations can co-exist with, and be independent from, other slower rhythms that arise in the same region.

It is also important to note that ripple characteristics (frequency, duration) in the full model receiving input from CA3 that was scaled in size saturated at around 80% of the baseline input and remained stable above this value (S4 Fig). Thus, ripple properties predicted by our model to match experimental data are observed in the broad range of CA3 mediated input amplitudes, i.e., structurally stable. Our results on the effects of reducing CA3 input on CA1 ripples are consistent with experimental observations made in simultaneous CA3-CA1 recordings [27], in which sizeable (hence synchronous enough) CA3 activity was related to CA1 ripples, while smaller (less synchronous) CA3 activity did not induce ripples in CA1.

### Role of pyramidal cells in ripple generation

After investigating the role of inhibitory spikes in ripples, we moved to study the role of pyramidal cells activity in our model. In fact, recent optogenetics work [21] has raised the interesting idea that the minimal circuit to obtain ripples in CA1 may include local excitatory synapses on inhibitory interneurons. Specifically, CA1 *in vivo* recordings showed that when activating both parvalbumin positive interneurons and pyramidal cells in the pyramidal layer, high-frequency oscillations (HFO) in the LFP emerged. When only pyramidal cells were driven optogenetically with a step of light, HFO were also measured, although their duration was shorter than HFOs obtained driving both neuron populations. In contrast, if only fast-spiking parvalbumin positive inhibitory interneurons in the pyramidal layer were driven to fire, LFP oscillations were not found. We set out to see if our model could show results consistent with these experiments.

**Fig 7:**
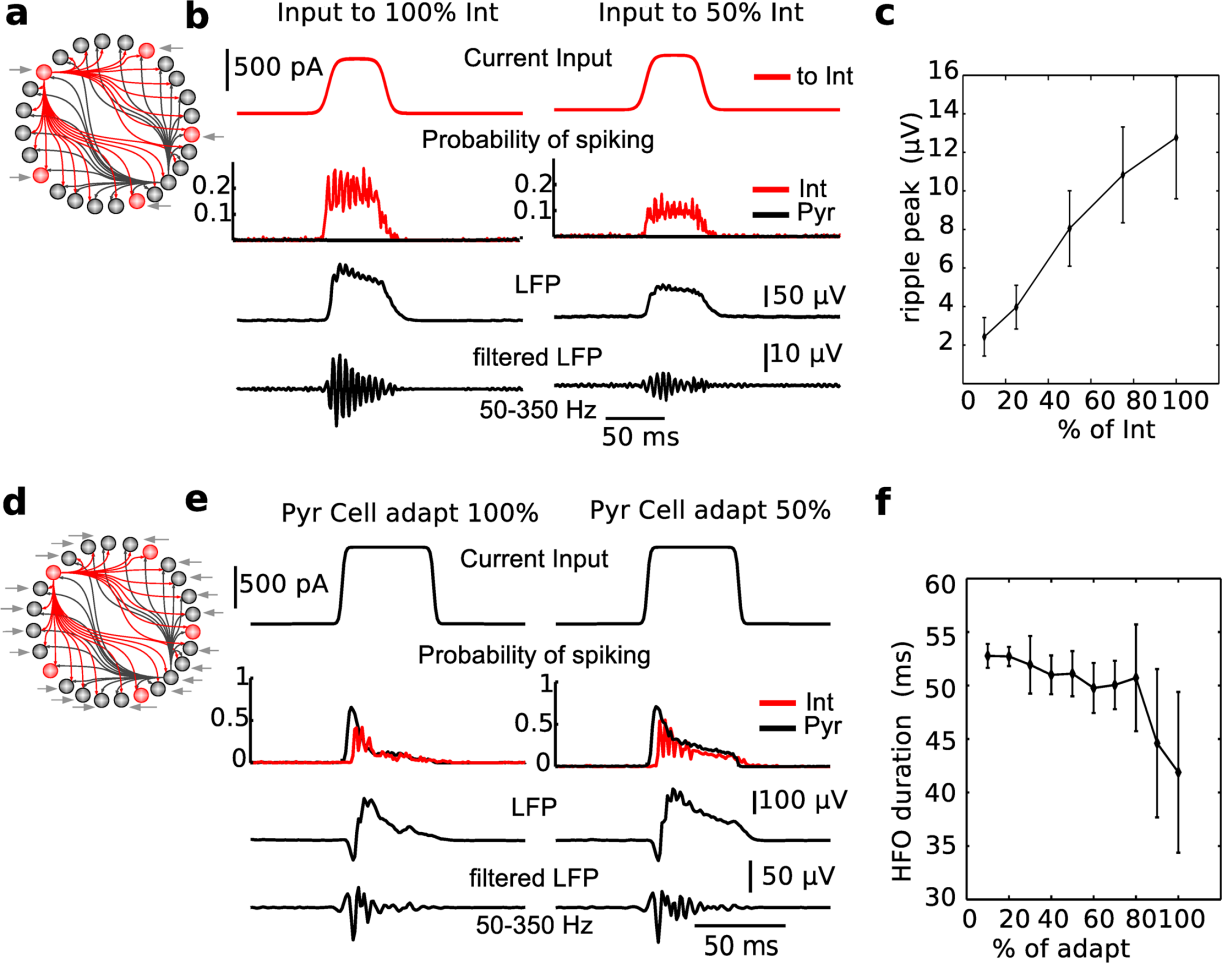
Effect of driving only Pyramidal cells or only Interneurons on ripple oscillations. (a) Schematic of the model, in which input current is delivered only to inhibitory interneurons. (b) Examples of model behavior. Top plot: input current. Middle plot: firing probabilities of interneurons (red) and pyramidal cells (black)–note that the pyramidal cells are not spiking during stimulation. Lower plots: LFP, wide band above and band-passed (50–350 Hz) below. (c) The ratio of interneuron receiving input current affects the size of filtered LFP. Note small amplitude of LFP when only a fraction of interneurons in activated. (d) Schematic of the model, in which only pyramidal cells receive input current. (e) Examples of model behavior. Same as in b. Note that reducing adaptation shows an increased duration of the high frequency event, which quickly (after less than 30ms) defaults to a gamma frequency oscillation. (f) Spike-frequency adaptation in pyramidal cells regulates the duration of HFO triggered by input only on pyramidal cells.

We started by delivering input current only to interneurons (Fig 7A): since simulations of a purely inhibitory network showed that current size controls synchrony among the interneurons (Fig 6A), the size of the current step was first kept the same as for the full model. In Fig 7B we show examples of spiking probabilities for two stimulation conditions (50% or 100% of all interneurons were stimulated) in the model when only interneurons received inputs. The inhibitory activity was still oscillatory, however all pyramidal cells were hyperpolarized, resulting in a field potential consisting of shunted currents. As a consequence, the LFP was much smaller than the one in the full model (compare Fig 7B with Fig 2D); LFP amplitude increased with the percent of interneurons recruited (Fig 7C). Specifically, in the case where 50% of interneurons were stimulated (which is likely much larger than any optogenetic stimulation *in vivo*), we found that the LFP amplitude was only about 10% of the LFP observed when both excitatory and inhibitory neurons received an input drive from CA3. Increasing the amplitude of current stimulation in the model led to even stronger shunting of pyramidal neurons and even smaller LFP amplitude. In comparing with experimental results, we emphasize that the reduction of the LFP amplitude in our model depends on shunting effect of inhibition on pyramidal neurons, which in actual *in vivo* experiments would depend on the cell geometry, location of excitatory and inhibitory synapses, and other factors that our minimal cell model cannot explicitly capture. Even in this simplified setting, we are able to show a qualitative match with the strong reduction of the amplitude of oscillations in the LFP. Thus, our model reveals that if only interneurons are driven by a light source, LFP oscillations will be very small in amplitude, regardless of the stimulation amplitude, to the point that they will be experimentally negligible, in agreement with *in vivo* results [21].

We next tested whether the input delivered only to pyramidal cells (Fig 7D) could give rise to short bursts of oscillations. Note that to achieve an initial spiking rate capable of triggering fast network oscillations, pyramidal cells had to receive a current step size bigger than the one used for the full model. Since light-driven cells in optogenetics experiments are not receiving a fixed step of current that is identical across populations, we chose to adjust the current size to the measured behavior. *In vivo*, the LFP initially showed high frequency oscillations (about 150 Hz), but only for about 25 ms [21]. Immediately after the initial high frequency response, the LFP amplitude in the high-frequency band decreased and the activity slowed down to about 80 Hz. In agreement with these data, in our model, we first achieved a high frequency firing for about 25 ms, but the pyramidal cell population was not able to sustain firing much longer (Fig 7E).

A variable controlling the ability of pyramidal cells to sustain firing is the strength of spike-frequency adaptation, which in the model equations is controlled by the size of the jump (b) imposed on the slow refractory variable (w) after each spike (see Materials and Methods). Since change in adaptation can be biologically achieved by a variety of neuromodulatory phenomena, and the less adaptive the cell is, the longer it could sustain firing, we tested whether the current input to pyramidal cells could generate ripple-like oscillations in the networks with progressively less spike-frequency adaptation in the pyramidal cell population. To reduce adaptation in our neurons, we multiplied the b value by a scaling factor, in a range 10–100%. We found that even when pyramidal cell firing can be sustained (Fig 7E), oscillations still showed the same profile of initial high frequency response that quickly slows down to a frequency below the ripple range as observed *in vivo*. Specifically, the amplitude of the LFP filtered in the high-frequency band (to detect the HFO) initially peaked but then dropped very quickly, which is consistent with what was shown *in vivo* (Fig 7F). We concluded that our model, in which CA3 input to both pyramidal cells and interneurons is necessary to trigger a ripple in CA1, is consistent with optogenetics data [21].

### Model predictions: Sequential activation of pyramidal cells in CA1 during ripples is mediated by sequential CA3 input

So far we have focused on a global mechanism of ripple generation, in which CA1 pyramidal cell timing within ripples is regulated by the ongoing rhythmic inhibition and incoming CA3 input. During sleep, CA1 pyramidal cells that spike in ripples are known to reactivate in firing order consistent with the one recorded during behavioral tasks [15, 26], and this property is thought to be a hallmark of a memory trace in the hippocampus. Memory trace reactivation has been measured in CA1, but it is not clear whether the mechanism inducing reactivation within ripples is intrinsic to CA1 or due to input. We set out to see which biologically relevant properties could control ordered pyramidal cell reactivation across ripples in our model.

It is known that neurons recruited by behavior to form a memory trace tend to have higher excitability [49–51]. We reasoned that cells in CA1 that reactivate during a ripple might have higher intrinsic excitability. Since, in our model, pyramidal cells are picking windows of opportunity to spike during ripples, cells with substantially higher DC levels will have an easier time finding a window of opportunity in which to spike (because overcoming the incoming inhibition would be easier for them, compared to all other cells). In our model, we tested whether all it takes to be replayed in sequence for a set of pyramidal cells activated during the training phase (and therefore known to have higher DC, and to spike in more ripples because of that) is the fact that they are more depolarized. If that was the case, than we would know that the sequential reactivation in our model results from competition between intrinsic cell depolarization and ongoing inhibitory oscillations.

It is also known that the Schaffer Collaterals (the projections from CA3 pyramidal cells to CA1 neurons) are plastic [52], hence potentially target-selective. In other words, neurons in CA1 could receive selective inputs from CA3: higher and more intense inputs from some CA3 pyramidal cells (presumably the ones correlated with the same behavior) and lower and less intense from other CA3 pyramidal cells. The selectivity of CA3 input could be further modulated by hippocampal inhibitory neuron types that are not modeled in our network, which are hypothesized to gate Entorhinal cortex input and CA3 input on CA1 pyramidal neurons [18, 53].

**Fig 8:**
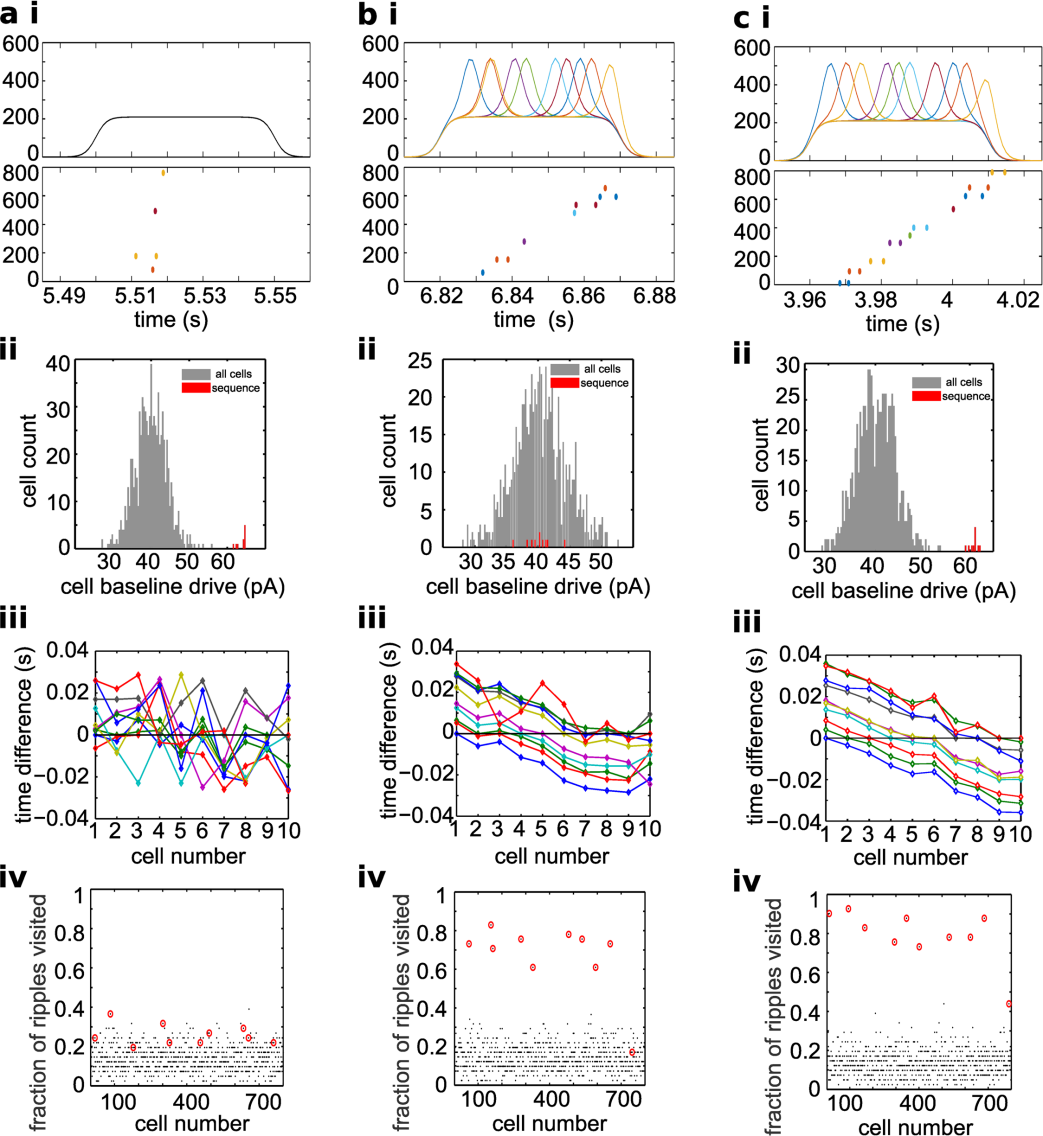
Selective input from CA3 induces sequential activation of CA1 pyramidal cells in model. (a i) Top panel, the CA3 input currents delivered to selected cells (sequence cells) during a ripple. Bottom panel, simultaneous rastergram of sequence cells. (a ii) Distribution of the input drives to all pyramidal cells in the model. In red, the values for sequence cells. (a iii) Average spike time differences for all sequence cells: each line is the average spike time difference between a given cell and all cells in the sequence, averaged across 40 ripples. The lines cross zero when the spike time difference with itself is reported. Note that in this case sequential spiking behavior during ripples is not present. (a iv) Plot of each pyramidal cell vs the fraction of ripples it visited. In black all cells, circled in red are the sequence cells. For every cell, the number of ripples in which it spiked (ripple visited) can be found and divided by the total number of ripples in the simulation. (b) Same plots as in a, but in this case selected cells only receive selective CA3 input (b i), without enhanced intrinsic excitability provided by constant direct current (see b ii). Note that in this case, the orderliness of spiking across ripples is overall preserved (see b iii). Note that most cells spike in less than 20% of the ripples, while selected “sequence” cells all visit about 70% of the ripples (see b iv). (c) Same as a, but in this case selected cells receive both enhanced intrinsic excitability (see c ii) and selective temporal ordering in CA3 input (see c i). The orderliness of the average spike time difference curves emphasizes that cells spike in order across multiple ripples (see c iii), and sequence cells spike in more ripples than all other cells (see c iv).

Hence we have two possible mechanisms with the potential of inducing reactivation: intrinsic CA1 excitability is a parameter of CA1 properties that could induce sequence reactivation, while CA3 selective input is a potential input-dependent mechanism for sequence replay in CA1. To test these two properties, we randomly selected 10 pyramidal cells (“sequence” cells) to represent neurons that reactivate sequentially during ripples. First, we increased the constant current input that the 10 selected neurons received (Fig 8A), which resulted in the appearance of a small peak at high values in the distribution of excitability of all pyramidal cells of the CA1 network (Fig 8A ii). This was introduced as a mechanism to increase the likelihood of selected cells to spike during ripples. In a second case, we changed the time course of incoming current input, only for the selected sequence cells (Fig 8B). As shown in Fig 8B–8I, each sequence cell received an input that had a peak in a narrow time window within the duration of a ripple. This peak represented the spiking of the sub-set of CA3 pyramidal neurons that were preferentially connected to the target CA1 cell; thus we assumed that there are CA3 neurons that would spike during a sequence-specific time window of the sharp wave event. In other words, in this case our model assumes that during a sharp wave-ripple there is an organized reactivation in CA3, inducing selective inputs to CA1, which results in sequential spike reactivation in CA1. Finally, in a third case, we combined both manipulations on our selected cells: increased excitability and selective CA3 inputs. For each case, we show whether the inputs from CA3 are selective in panel i, and whether sequence cells received extra excitability in panel ii.

In our model, sequence replay needed to lead to two properties: (a) specific spike sequences repeated more often than chance and (b) the temporal order of the spikes of CA1 cells within those sequences was consistent across ripples. For the replay properties to be satisfied, we checked if our sequence cells spiked in a greater fraction of ripples compared to all other cells (panel iv), and spiked in a consistent order across ripples (panel iii). To verify that, we computed the spike time difference between sequence cells, and averaged across all ripples in a 10 s simulation (41 ripples). If the order of cells was maintained across ripples, the average spike time differences will look like shifted versions of the same line.

Fig 8 shows the effect of these manipulations. In Fig 8A, selected cells only received enhanced intrinsic excitability (see a ii) without selective temporal ordering in CA3 input (see a i). Note that, in this case, there was no sequential spiking behavior during ripples (see a iii), and sequence cells did not spike in more ripples than all other cells (see a iv). In Fig 8B selected cells only received selective CA3 input (see b i), without enhanced intrinsic excitability provided by constant direct current (see b ii). Note that, in this case, the orderliness of spiking across ripples was overall preserved (see b iii). Also note that the fraction of ripples visited (b iv) was higher for sequence cells compared to all other cells. In Fig 8C we show that CA1 neurons that received selective input from CA3 (c i) and higher intrinsic excitability (c ii) showed spiking in a greater fraction of ripples compared to all other cells (c iv), and spiked in a consistent order across ripples (c iii). Note that compared to condition b, in which the additional intrinsic excitability is not present, the consistency of ordered firing across ripples is improved.

In summary, we found that being more depolarized served the selected cells well in terms of the number of ripples during which they spike, but not for spiking in the orderly fashion that is found experimentally. On the other hand, selective CA3 input seemed to be effective at inducing sequence reactivation. The necessary elements of the model to allow for mapping of the structure in CA3 input onto spiking in CA1 pyramidal cells were 1) generalized input lasting throughout the sharp wave event to most cells in CA1; 2) time-selective, ordered input from some CA3 cells to some pyramidal cells in CA1; 3) the selective input, when present, results in current delivered to its target (sequence) cells at higher magnitude than generalized input delivered to the non-sequence cells (because of plasticity in CA3-to-CA1 synapses). Our model therefore predicts that replay in the hippocampus is generated in CA3 (and possibly in the dentate gyrus) and ordered reactivation in CA3 is required for reliable sequential spiking in CA1, where it is packaged in a fast-rhythm at rates that are conducive to synaptic plasticity, to be transmitted to target regions, such as the cortex. Intrinsic CA1 properties, such as heightened excitability of selected cells, can enhance but not cause the ordered replay.

## Discussion

Synchronized neuronal activity manifested by different types of EEG and LFP rhythms takes over cortex and hippocampus across sleep stages. During non-REM sleep, cortical slow oscillations alternate between highly active phases (Up states) and Down states, in which most cells are hyperpolarized, at very low frequencies (0.2–1 Hz) [8]. In the hippocampus, sharp wave-ripples (SWR) are brief high-frequency (>150 Hz) events that have a temporal relationship with transitions to Up states during slow oscillations [16]; and which might be crucial to sleep-dependent memory consolidation [12, 13, 54]. Moreover, hippocampal reactivation, a process in which cells active during a task fire during subsequent sleep in a task-consistent order, is known to take place within SWR [15]. Replay has also been found in cortex [55], and it has been shown to co-occur in visual cortex and hippocampus during sleep [56]. Despite the important role of SWR in the interplay of hippocampus and neocortex during memory consolidation, neuronal mechanisms underlying the process of ripple generation are still largely unknown [20, 57].

In this study, we propose a novel mechanism of hippocampal CA1 ripple generation consistent with a broad set of experimental data. We design a model of CA1 where synchronization of interneurons is due to common input from CA3, rather than reciprocal inhibition, and where inhibition regulates the windows of opportunity for pyramidal cells to spike. Our *in vivo* data show that ripples have a broad frequency distribution but very small variability in duration, which cannot be explained by existing models of high frequency oscillations. Our study predicts that ripple duration is constrained by the CA1 architecture; specifically that it depends on the transient nature of the synchronization of inhibitory neurons driven to fire at high frequencies by incoming excitation from CA3 in the presence of noise. Our model makes testable predictions on the effect of manipulating of GABA_A_ on ripple frequency and percent of pyramidal cells recruited.

### Mechanisms of ripple

In general, there is an agreement in the field that parvalbumin positive interneurons are involved in LFP ripple oscillations, since they spike at ripple frequency across the event duration [31] and removing GABA_A_ cancels ripple activity [29]. Differences arise in the interpretation of the specific mechanism underlying the oscillations. Two main hypotheses have been proposed. According to one idea, a ripple in CA1 is exactly the same phenomenon as its triggering CA3 excitatory event, simply propagating from one hippocampal sub-region to the next. In this setting, a ripple can be thought of as fundamentally one whole excitatory event reverberating across the hippocampus, much like throwing a small stone in still water [17, 58]. Models that account for such behavior have to include non-standard excitatory mechanisms among CA1 pyramidal cells, such as more-than-linear dendritic summation [58] or electrotonic connections (known as gap junctions) [17, 59, 60]. However, recent *in vivo* recordings from a strain of gap-junction deficient mice still showed sharp wave-ripple events at frequencies similar to that of the wild-type [61, 62]. This led to the idea [19, 53, 57] that it is appropriate to introduce models in which CA1 synapses can generate ripples. According to this approach ripples are seen as a local phenomenon in CA1 triggered by incoming CA3 excitation [18, 19, 57], in which inhibitory synaptic connections are responsible for the oscillations in CA1. Our model is consistent with the latter hypothesis. We propose that the minimal model capable of ripple generation is an inhibitory network receiving a brief wave of excitation. The crucial role of parvalbumin positive basket cells in organizing ripple oscillations has been previously shown by Schlingloff et al [46], who used a network of only parvalbumin positive interneurons to study ripple frequency when a step of current was applied to the population. While in this earlier model the loss of reciprocate inhibitory synapses induced a loss of rhythmicity, we now show that oscillatory firing in such inhibitory networks is controlled by a synchronous and strong common input, which is characteristic of a transient oscillation. The strength of recurrent connectivity between inhibitory interneurons plays a critical role in determining the type of oscillatory activity the inhibitory network can produce. Indeed, if synaptic connections were strong, a purely inhibitory network with enough reciprocate connections would give rise to gamma oscillations (30–90 Hz), paced by the duration of inhibitory currents: a mechanism known as Interneuron-Network Gamma (ING) [63, 64]. ING oscillations persist as long as cells are driven to fire. In contrast, the stationary behavior of our model is a disorganized firing state. Oscillatory persistent firing in a network of irregularly spiking inhibitory neurons depends on synaptic strengths and the size of noise [65]: our model belongs to the asynchronous stable state part of the bifurcation diagram (Fig 5A in [65]), where the size of noise overcomes the ability of mutual inhibitory synapses to organize the firing rate in oscillations that would be below ripple frequency. Crucially, we assume synapses to be weak, meaning that inhibitory currents do not overcome the effect of intrinsic noise even when a lot of interneurons are spiking. Hence, interneurons are driven to fire by CA3 inputs and not slowed down by inhibition. As a consequence, the ING mechanism is not arising in the network, and inhibitory currents are not setting the firing frequency, but merely modulating it. Instead, upon input arrival, inhibitory interneurons become transiently synchronized, leading to high frequency LFP oscillations, with properties matching *in vivo* data; the synchronization then disappears after a characteristic duration in the presence of noise. While the minimal mechanism is identified in a purely inhibitory network, we emphasize that transient oscillations in that reduced model did not last longer than 40ms. This underscores the role that pyramidal cells and their interaction with interneurons still play in shaping a realistic ripple oscillation in the full model we present.

Our model predicts that synchrony of interneuron firing should decrease over the ripple duration. Spontaneously occurring ripple-like activity in slices suggest that during a ripple excitatory and inhibitory currents onto pyramidal cells oscillate at ripple frequency and increase in size during the progression of a ripple [66], which likely reflects the increasing number of cells involved in the slice spontaneous event, rather than a higher synchronicity in cell firing. On the other hand, optogenetically induced ripples *in vivo* and *in vitro* seem to have a number of similar properties, and *in vivo* light-triggered events are likely to account for spontaneous, physiological ripple oscillations. Hippocampal slices in which ripples are triggered with optogenetic drive show that synchrony among nearby inhibitory neurons decreases across a ripple event [46].

### Beyond CA1 and minimal model of ripple generation

Beyond parvalbumin positive basket cells, different hippocampal interneuron types show different spiking behaviors during ripples [30, 31, 43, 67], and they might be involved in the fine timing of specific pyramidal cell spiking or recruitment, and in gating entorhinal and thalamic inputs on CA1 pyramidal cells. Furthermore, post-inhibitory rebound in pyramidal cells has been proposed as a mechanism for ripple initiation in CA3 [57]. Previous models have been trying to dissect potential separate roles for different interneuron types in gating entorhinal and CA3 input on CA1 and potentially contribute to fine selectivity of the pyramidal cells recruited by a given ripple [53]. The goal of our study was to find a minimal model capable to explain ripple oscillations frequency and duration in CA1, therefore we did not model the multitude of interneurons beyond parvalbumin positive basket cells. We focused on the pyramidal layer and its main spiking actors during the events our model represents. We emphasize that in previous modeling approaches [17, 19, 58] ripple duration is established by the duration of excitatory propagation within CA3. We show here that this does not have to be the case. In fact, we predict that the CA1 network is responsible for determining the duration of the organized firing during ripple oscillations.

The origin of the excitatory CA3 event which initiates a ripple in CA1 is not yet clear [46], and goes beyond the scope of this work. We predict that such CA3 events could show a broad range of durations, but they will still induce ripples of a fixed length, lasting about 50–80 ms in CA1. This implies that the CA1 region can produce a standardized “package” of information with every ripple, which is projected to downstream regions. Within this package, pyramidal cells spiking is organized by selectivity of CA3 input, and possibly input directly from other brain areas. We also predict that pyramidal cell replay in CA1 is organized by pyramidal cell replay in CA3, consistent with the fact that CA3 to CA1 connectivity is crucial for memory consolidation [54].

A common hypothesis is that synaptic connections between CA1 pyramidal cells and intrinsic cell properties regulate the order of spiking in a CA1 sequence [53]. In our study we tested this hypothesis first. Since in CA1 very few pyramid-to-pyramid synapses are found, we only had to check the effect of DC input. We found that preferential DC levels, even much stronger than average, were not enough to guarantee a robust repetition of the correct order of firing in our target cells. We then tested the potential role of the incoming CA3 input. Given the projection onto CA1 pyramidal cells are plastic, and that CA3 is the most typical set in which Hebbian learning is found in the brain, we formulated the hypothesis that CA3 is replaying its own cells, and projecting selectively to CA1 the activity that is relevant to behavior, because cells in CA3 and CA1 were active together during behavior (and hence learning). We then found that a combination of the two factors was best at inducing hippocampal replay in CA1 in our model.

Our study further predicts that when activating only a fraction of basket cells, the mechanism for ripple oscillations is present but not measurable in the LFP at the stimulation location. In fact, pyramidal cells would be shunted by an incoming barrage of inhibition and would not be sustained by excitation. This results in a shunted LFP in the CA1 pyramidal layer. While our minimal model does not show an exact quantitative match with experiments, our representation of the effect of optogenetically driving only interneurons results in a strong reduction of the magnitude of the LFP, which we believe is consistent with what has been found in published data [21]. Our model is also consistent with experimental results on optogenetic stimulation of only CA1 pyramidal cells. Both data and our model show the initial high frequency events lasting about 25 ms were quickly followed by oscillations at a slower frequency. This means that ripple generation *in vivo* has to rely on additional mechanisms other than excitation of pyramidal cells to sustain firing beyond 25–50 ms. We suggest that the CA3 input driving both the excitatory and the inhibitory populations in CA1 pyramidal layer raises the firing rate of basket cells to within ripple frequency range, inducing transient oscillations, while maintaining the excitatory population above shunting level, so that selected pyramidal cells can fire during ripples, and so that their spike timing stays modulated by ripple phase.

In our model, input from CA3 activates both CA1 interneurons (hence triggering ripple activity) and CA1 pyramidal cells. The selection of which pyramidal cell is recruited to spike within a given ripple, and their potential sequential activation, is the result of a balance between CA3 drive, (potentially filtered by different types of interneuron populations omitted in the model [18, 53]) and local feedback inhibition in CA1. Hence, CA3 is seen as the place in which the initial reactivation of a memory can take place, while CA1 optimizes CA3 output for downstream transmission. In other words, ripples in CA1 prepare a time-bounded package of carefully selected pyramidal cell spikes, encoding information that can then be routed across neocortex. It is also possible that incoming excitatory input from entorhinal cortex could be responsible for initiating a CA1 ripple phenomena, and/or regulate the recruitment of CA1 pyramidal cells within a ripple. In fact, this computational model represents CA1 as a network which is ready to burst, just waiting for an incoming input to select a local group of interneurons to oscillate, so that they can organize the timing of pyramidal cells, recruited by the input combined with their initial excitability.

## Materials and Methods

### Data collection and analysis

Data were recorded using extracellular tetrodes targeted to region CA1 of the hippocampus in 6–7 months old Brown Norway rats during natural sleep. All experiments were approved by the University of Arizona IACUC and followed NIH guidelines. The recorded LFP where band-passed between 0.1 and 500 Hz, and SWR complexes were found when the filtered LFP (100–300 Hz) crossed a threshold of 2 standard deviations of the baseline. The center of the SWR was positioned at the peak values of the LFP envelope and SWR start and end were found as the first points around the peak where the envelope passed below a threshold of half the distance between the peak and the baseline, see S1 Fig for a schematic. Ripple duration was then computed as the time between SWR start and end. Note that discrepancies between the numbers reported for ripple duration in our work compared to other reports could be induced by different methods of finding ripple durations, beyond potential differences between rats and mice. In our method of detecting ripple starts and ends, we use as a threshold the half distance between baseline and peak. That is, every ripple has its own threshold, which depends on the size of the ripple amplitude. If one were to use one absolute threshold for all ripple envelopes to find their start and end time points, ripples that have larger peak amplitudes would stay longer above threshold, and the average value of ripple duration would be larger as a result. This difference in methods can contribute to the apparent difference in the data across the many ripple papers in the field. Ripple frequency was calculated as the average inter-peak interval of the filtered LFP within the SPW duration, see S1 Fig for a schematic. The Gaussian fit for the frequency distribution was found using MATLAB (www.themathworks.com) function fitdist. Ripple inter-arrival times are simply the time difference between a ripple onset and the next. The exponential fit for the distribution was obtained using function expfit in MATLAB. Data is presented as standard deviation from the mean.

### Computational model–equations and parameters

The CA1 computational model includes 160 interneurons and 800 pyramidal cells. For each neuron, the equations are

$$C\;\dot{v}=-g_{L}(v-E_{L})+g_{L}\Delta \;exp\left(\frac{\left(v-V_{t}\right)}{\Delta }\right)-w+I\left(t\right)$$

$$\tau_{w}\dot{w}=a(v-E_{L})-w$$

$$v(t)=V_{thr}\Rightarrow \;v(t+dt)=V_{r},\;w(t+dt)\;=\;w(t)+b$$

Parameter values are as follows. For all pyramidal cells: C = 200 pF; g_L_ = 10 nS; E_L_ = -58 mV; a = 2; b = 100 pA; Δ = 2 mV; τ_w_ = 120 ms; V_t_ = -50 mV; V_r_ = -46 mV, V_thr_ = 0 mV. For fast spiking inhibitory interneurons: C = 200 pF; g_L_ = 10 nS; E_L_ = -70 mV; a = 2; b = 10 pA; Δ = 2 mV; τ_w_ = 30 ms; V_t_ = -50 mV; V_r_ = -58 mV.

Every cell is receiving a different input, all with the same structure:

$$I\left(t\right)=\;I_{DC}+\beta \eta_{t}+I_{syn}\left(t\right)+I_{inp}\left(t\right)$$

$$\tau \;d\eta_{t}\;=\;-\eta_{t}\;dt+dW_{t}$$

$$I_{inp}\left(t\right)={I_{max}\left(1+\text{exp}\;\left(-\frac{t-t_{on}}{k}\right)\right)}^{-1}{\left(1+\text{exp}\;\left(\frac{t-t_{off}}{k}\right)\right)}^{-1}$$

Where *I*_*DC*_ is a constant, different for each cell, selected from a normal distribution (mean 40 pA for pyramidal cells and 180 pA for interneurons; standard deviation 10% of the mean for both populations). η_t_ is a stochastic process known as Ornstein-Uhlenbeck (OU) process, with a filter time scale imposed by the τ value, in our case 100Hz. The coefficients were β = 80 for pyramidal cells, β = 90 for interneurons. To computationally introduce a stochastic process that solves the equation for η_t_ above, we first generated its representation in the frequency space taking advantage of its known power spectral density [68], and then computed its inverse Fourier transform (ifft in MATLAB, www.themathworks.com).

Synaptic currents are modeled with double exponential functions, for every cell *n* we have $Isyn\;\left(t\right)=\sum_{j=1}^{160}g^{j\to n}\;s^{j\to n}\left(t\right)(v_{n}-E_{i})+\sum_{j=1}^{800}g^{j\to n}\;s^{j\to n}(t)(v_{n}-E_{e})$

$$s^{j\to n}\left(t\right)=\sum_{spikes\;of\;cell\;j}F\;\left(e^{H\left(-\frac{t-t_{k}}{\tau_{D}}\right)}-e^{H\left(-\frac{t-t_{k}}{\tau_{R}}\right)}\right)$$

with *F* a normalization factor that ensures at every spike the double exponential peaks at one, *H(·)* is the Heaviside function. Every *g*^*j -> n*^ is selected from a Gaussian distribution with a given mean and standard deviation 10% of the mean (see Fig 2); values below 0 are rectified to 0. Mean synaptic values are ${\bar{g}}_{Int\to Int}$ = 0.0234 nS, ${\bar{g}}_{Pyr\to Int}$ = 0.0083 nS, ${\bar{g}}_{Int\to Pyr}$ = 0.0521 nS, ${\bar{g}}_{Pyr\to Pyr}$ = 0.001 nS. The reversal potential for synaptic currents are *E*_*i*_ = -80 mV and *E*_*e*_ = 0 mV. The time scales of synaptic rise and decay are as follows: for excitatory synapses on pyramidal cells [34, 39] we have τ_R_ = 0.5 ms and τ_D_ = 3.5 ms; while on interneurons we have τ_R_ = 0.9 ms and τ_D_ = 3 ms. For inhibitory synapses on interneurons: τ_R_ = 0.3 ms and τ_D_ = 2 ms, on pyramidal cells τ_R_ = 0.3 ms and τ_D_ = 3.5 ms.

*I*_*inp*_*(t)* represents the net effect of CA3 synaptic excitation on CA1 cells. t_on_ and t_off_, in most simulations are kept 50ms apart. *I*_*max*_ = 210 pA for pyramidal cells and *I*_*max*_ = 700 pA. Network simulations were solved with a 1-step Euler algorithm (Δt = 0.001 ms) using MATLAB. (www.mathworks.com)

### Computational model–rationale

In this section we introduce the rationale behind the choices of equations and parameters used to model CA1 ripple activity and CA3 input to CA1.

We model only the pyramidal layer of CA1, because that is where ripples are usually measured. To reveal the basic mechanisms of ripple generation, we do not model the rich number of interneuron cell types known to exist in CA1, but only the parvalbumin positive basket cells, which are active during ripples [32] and are a predominant interneuron type in the pyramidal layer. Ripple oscillations are only seen in the pyramidal layer [24], so we had to include at least the populations known to be dominant in such layer: pyramidal cells and fast-spiking interneurons. While a rich number of interneuron cell types are known to exist in CA1, we thought that they were going to have limited impact on ripples, for the following reasons. Bi-stratified cells are known to spike during ripples, hence they could potentially also contribute, but they project on the proximal dendrites of pyramidal cells, while basket cells project at the soma. This means that synaptic events due to basket cells spiking will have bigger representations and effect on pyramidal cells voltage, and ultimately spikes. Another cell type known to be important to hippocampal theta rhythm, the OLM cells [69], might be relevant outside (that is, before and after) ripples. OLM cells spike only in about half the ripple episodes in naturally sleeping animals [23], and transmit inhibition to pyramidal cells via slower IPSCs (longer than 10ms time scale [70]). This inhibitory time scale, together with their intrinsic oscillatory properties, makes OLM cells amazing candidates in the participation to hippocampal theta rhythms [69, 71–73]; however, ripples are fast oscillatory events caused by incoming CA3 signals: the role that OLM cells could reasonably play in such events (given they do not spike during half of them) is potentially modulate the amount of excitation it would take to a CA3 input to start a ripple. In other words, they could be contributing to switching from ongoing theta activity to ripple activity in awake state [74]. Since our is a model of what happens when the sharp wave activity from CA3 hits CA1 and therefore fast oscillations arise, we are not modeling the switch between theta and other rhythms. Therefore, we consider it acceptable to omit the modeling of OLM cells activity during ripples in our model. We emphasize that if this was a model of awake state, in which ongoing theta-gamma oscillations are interspersed with sharp wave-ripples, modeling of the activity of these interneuron types would be necessary.

In our model, we only have parvalbumin positive basket cells to represent the overall disorganized background inhibition that matches and balances the ongoing excitation. Hence, when we set up the ratio of pyramidal cells to interneuron, we choose a ratio that encompasses the overall excitatory to inhibitory ratio in CA1, rather than the fine count of basket cells. We also model pyramidal cells as one uniform population even if it is known that they are quite selective in their specific connectivity, because we are interested in representing the overall oscillatory phenomena more than the specific cell-to-cell variability. The proportion of interneurons and pyramidal cells in the model is in agreement with CA1 anatomy [32]. We choose to model both neuron types with Exponential Integrate and Fire equations, because of its simplicity (only two variables) combined with a formalism that can represent explicitly spike-triggered adaptation and intrinsic cell resonances (essential to give CA1 pyramidal cells their characteristic bursting spiking profile [34]). In looking for appropriate parameter values for the model cells types, we build on work by Gerstner [36], who has classified parameter sets for the Adaptive Exponential IF model that match known spiking behaviors such as bursting (for pyramidal cells) and regular fast spiking (for basket cells). We chose parameters that guaranteed a time scale (see C/g_L_ ratio) for membrane voltage of about 20ms for both cell populations, the ability of pyramidal cells to burst when driven to fire (the value of V_r_ is crucial for that), a theta resonance in pyramidal cells given by τ_w_, and the regular spiking behavior of basket cells. Given that all cells of a population are modeled by the same equation, we introduce heterogeneity in the network using input currents and variability in synaptic strengths.

Special attention was made to design an input term *I(t)* that is composed of different parts. The *I*_*DC*_ term variability produces variability in the excitability level of each cell, therefore introducing a first level of heterogeneity in the network. The noise term *βη*_*t*_ represents the *in vivo* state of the voltage in each cell, which is likely receiving a much higher barrage of synaptic inputs than the one provided by the network spiking activity. *η*_*t*_ is an Ornstein Uhlenbeck (OU) process, which can be thought of as a filtered white noise process. This kind of noise does not introduce slow frequency forcing, which could alter the network behavior, or too high frequency voltage fluctuations, which are known to not be present [75]. This kind of noise is also used in dynamic-clamp experiments to mimic *in vivo* state in hippocampal slice recordings [38]. The β coefficients establishing noise size were chosen so that hyperpolarized cells show a voltage fluctuation of about 2mV in size [38, 75].

*I*_*syn*_ is the term representing synaptic current in the input. With the chosen formalism for synaptic equations, the strength of synaptic connections g^j→n^ scales the maximum peak size of a post-synaptic potential. Every g^j→n^ is selected from a Gaussian distribution to introduce heterogeneity. The reversal potential for synaptic currents are chosen in agreement with many published hippocampal models [34], and the time scales of synaptic rise and decay are estimated from literature [34, 39], in particular taking advantage of the fact that EPSP on interneurons are faster than on principal cells [76] and IPSPs from basket cells are slower on pyramidal cells than on other basket cells [39]. Average synaptic strength values in the model induced post-synaptic potentials of less than 1mV and have been tuned to induce a balanced average of excitatory and inhibitory currents in pyramidal cells. Thus we applied a common approach of normalizing the average synaptic weight by total number of cells in each population [77], hence taking into account the fact that the total incoming excitatory connections in a given cell are more than the inhibitory ones (note the magnitude of ${\bar{\text{g}}}_{\text{Int}\to \text{Int}}$ compared to ${\bar{\text{g}}}_{\text{Pyr}\to \text{Int}}$). The synaptic values we chose resulted in small post-synaptic potentials, where a single or few synchronous incoming spikes are not enough to cause a spike or suppress one in the post-synaptic cell. This condition implies the network is weakly coupled [78] as a dynamical system.

The incoming current input *I*_*inp*_*(t)* is bell-shaped, gradually rising and falling, between t_on_ and t_off_. Incoming input from CA3 reaches a maximum value *I*_*max*_ different across populations, chosen to obtain a fraction of pyramidal cell spiking during ripples consistent with experimental data [24, 43] and a firing frequency for interneurons about 120 Hz [23]. When a net current is used to represent the sum of a barrage of incoming post-synaptic currents, such current has to take into account the scaling that cell properties will operate on the post-synaptic currents. Since cell membranes have capacitive and resistive properties, there will be a time scaling by a time constant which is the cell time constant. Since we add the current input to the right-hand side of the dv/dt equation, this filtering is operated by solving for voltage in time. The other filtering that cells can operate is due to their size. Input resistance is defined as the ratio between difference in voltage induced by a step of current and the size of the current step. As such, it interacts with cell size: more current is required to change the voltage of a larger cell. Our model cells are point cell, that is, they do not have a radius. We have not defined the overall cell parameters based on a per-area scaling, but rather on their dominant time-scale properties. As a result, the intuitive choice of giving to both pyramidal cells and inhibitory interneurons the same size of current step (because the same CA3 cells are connected to them) would be overlooking the well-known fact that pyramidal cells are much bigger cells that parvalbumin positive interneurons. Rather than re-scaling the model to introduce size properties, we re-scale the incoming current.

### Modeling the LFP

While the exact nature of the LFP components likely includes both synaptic activity and spikes [79], we here focus on the CA1 pyramidal layer, where perisomatic interneurons are known to synapse, and are interested in a phenomena in which pyramidal cells spikes are known to not contribute significantly. To obtain an LFP estimate, the average synaptic current input across all pyramidal cells was calculated, and then rescaled by 1 mS to represent a potential, such that 100 pA of synaptic current produce a 100 μV LFP change. In the model, SWR were found when the filtered LFP (50–350 Hz) exceeded a threshold of 5 standard deviations of the mean computed in one SWR-free second of activity.

### Analysis of inhibitory network model

To estimate the duration of synchrony in a purely inhibitory network receiving a current step input of size k at time t = 1s (Fig 5), we considered histograms of spike probability constructed averaging 200 trials. The asymptotic value (lim) of the histogram was the average firing probability between 1.5 s and 2 s. The heights of peaks in the histograms were decreasing in time. The size of the first peak (p1) defined a threshold 0.2*(p1-lim). The first peak that was far from lim less than threshold marked the end of the transient.

## Supporting Information

S1 Fig Details of ripple property estimation. The black line represents a filtered trace (in our case 100–300 Hz), z-scored. The blue line marks the threshold (in our case, half the distance between the peak and the baseline). Arrows mark peaks of ripple oscillations, not that the peak of a ripple event is found where the LFP amplitude is maximized.
(TIF)

S2 Fig Statistics of ripple properties in CA1. For each ripple in a 25hr in vivo experiment, its frequency is plotted against the time to the next ripple (a). Its duration is plotted against the time to the next ripple (b). Note that there is no clear correlation between the time to the next ripple and current ripple frequency or duration. Poincare return maps relate a property of the current ripple (n-th ripple) to the same property (or a different one) in the next ripple (n+1-th ripple). If the system has memory, then a property of a ripple is predictive of the same (or a different) property in the next ripple. In a system with no memory, the cloud distribution should look like the direct product of the distributions of the two properties considered. (c) Ripple frequency does not show an obvious memory effect. Note that the distribution in n vs n+1 looks like the direct product of Fig 1B with itself. (d) Ripple duration does not show memory effect across ripples. Compare with Fig 1D times (outer product) itself. (e) Current ripple frequency does not influence next ripple duration. (f) Current ripple duration does not affect next ripple frequency.
(TIF)

S3 Fig Autocorrelation of firing probability of interneurons (red) and pyramidal cells (black) shows no background frequency properties in the network. (TIF)

S4 Fig Changing the magnitude of CA3 input affects ripple amplitude, frequency and duration. (a) Example of a simulation in which CA3-mediated input current in both pyramidal cells and interneurons of the CA1 model is reduced to 30% of its baseline magnitude, by multiplying *I*_*inp*_*(t)* by 0.3. Note the size of y-axis on the top panels. The right column shows a smaller time interval, so that the “ripple” profile can be seen. Time is in seconds in all panels. Top panels: current input (in pA) from CA3 to pyramidal cells (black) and interneurons (red). Second panels from the top: rastergram of pyramidal cells (black) and interneuron (red) spikes. Middle panels: probability of spiking for pyramidal cells (black) and interneuron (red) populations, in 1ms time bins. Last two panels: wide band (above) and filtered (100–300 Hz) LFP trace (in μV). (b) Same as in (a), but for CA3-mediated current only scaled to 80% of its baseline strength. Note how the interneuron population fires more organized, which results in a filtered LFP more structured in this case, compared to the 30% scaling. (c) Summary plot of core ripple properties when the input from CA3 to both pyramidal cells and interneurons is scaled in a range of 10–100%. Ripple amplitude rises from 5 μV (undetectable) as input size increases, and saturates between 80–100% of the input. Ripple duration is ill-defined at 10% input (note the great variability as a number of events that qualify for ripple detection do not show enough oscillations for the duration to be consistently estimated), and increases with increasing input amplitude. Ripple frequency is over-estimated below 30% due to the 100–300 Hz filtering in ripple detection, but once input is above 30% one can see the shift from high-gamma to ripple range, controlled by input size.
(TIF)

S5 Fig Network activity without I-to-I synapses. (a) Example of a typical ripple event in the network when I-to-I synapses are removed. Top: input current (in pA) from CA3 to pyramidal cells (black) and interneuron (red) population. Middle: rastergram of pyramidal cells (black) and interneurons (red) spikes during a ripple. Lower plot: wide-band (black) and filtered (100–300 Hz, red) LFPs in the network. Note that the oscillations stop much quicker than in the network with I-to-I inhibition shown in Fig 2. (b) Summary histograms for ripple frequency (Hz), duration (ms) and amplitude (μV) in the case of removed I-to-I synapses. The overall properties of ripples are on average preserved (as expected), yet the filtered LFP is unable to ever generate ripples longer than 60m, compare with Fig 3B.
(TIF)
