# Supplementary figures and images for: Hippocampal CA1 Ripples as Inhibitory Transients
Source: PLoS Comput Biol. 2016 Apr 19;12(4):e1004880. doi: 10.1371/journal.pcbi.1004880 (PMC4836732; doi:10.1371/journal.pcbi.1004880)

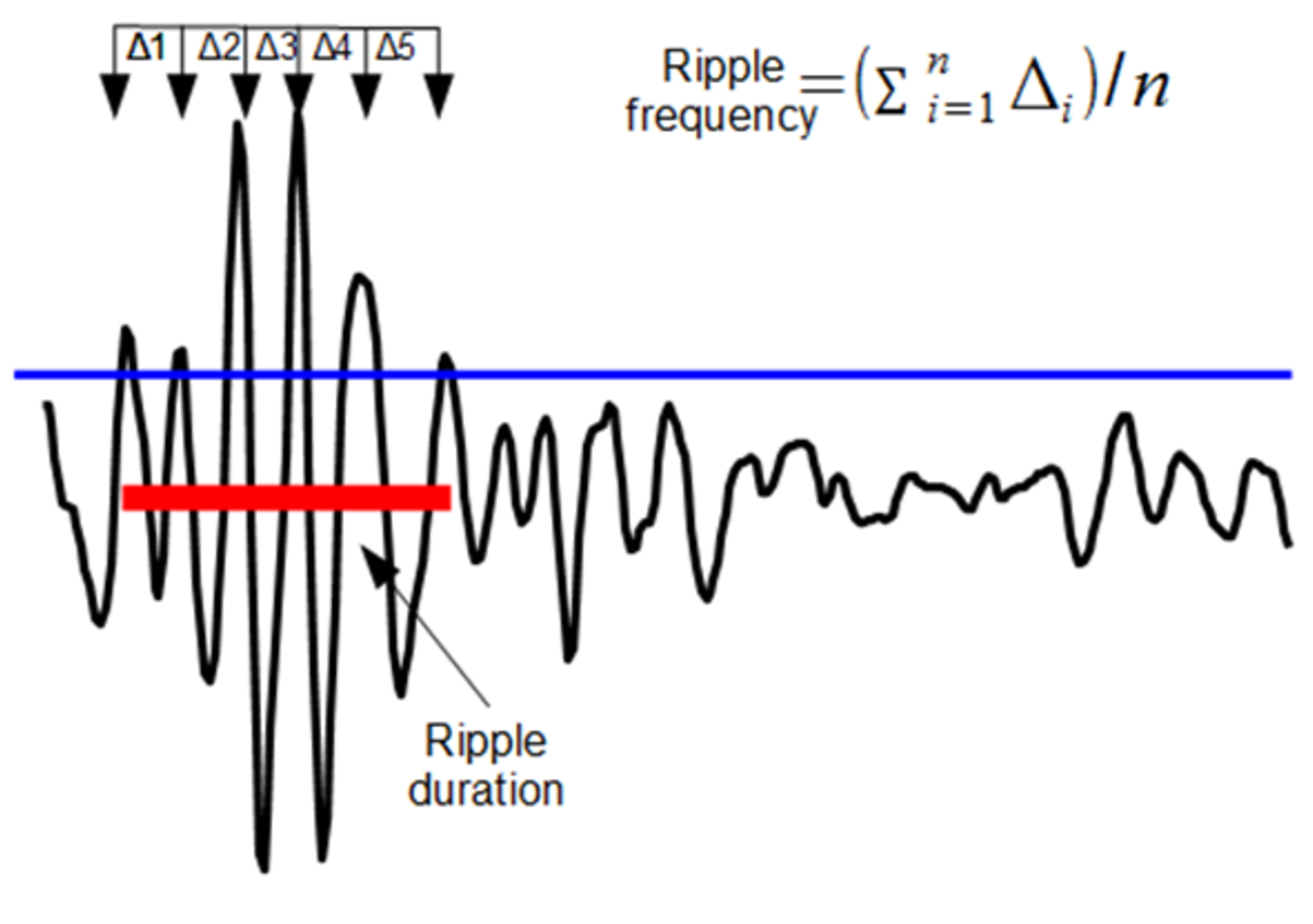

Supplement: S1 Fig — The black line represents a filtered trace (in our case 100–300 Hz), z-scored. The blue line marks the threshold (in our case, half the distance between the peak and the baseline). Arrows mark peaks of ripple oscillations, not that the peak of a ripple event is found where the LFP amplitude is maximized. (TIF) [file pcbi.1004880.s001.tif]

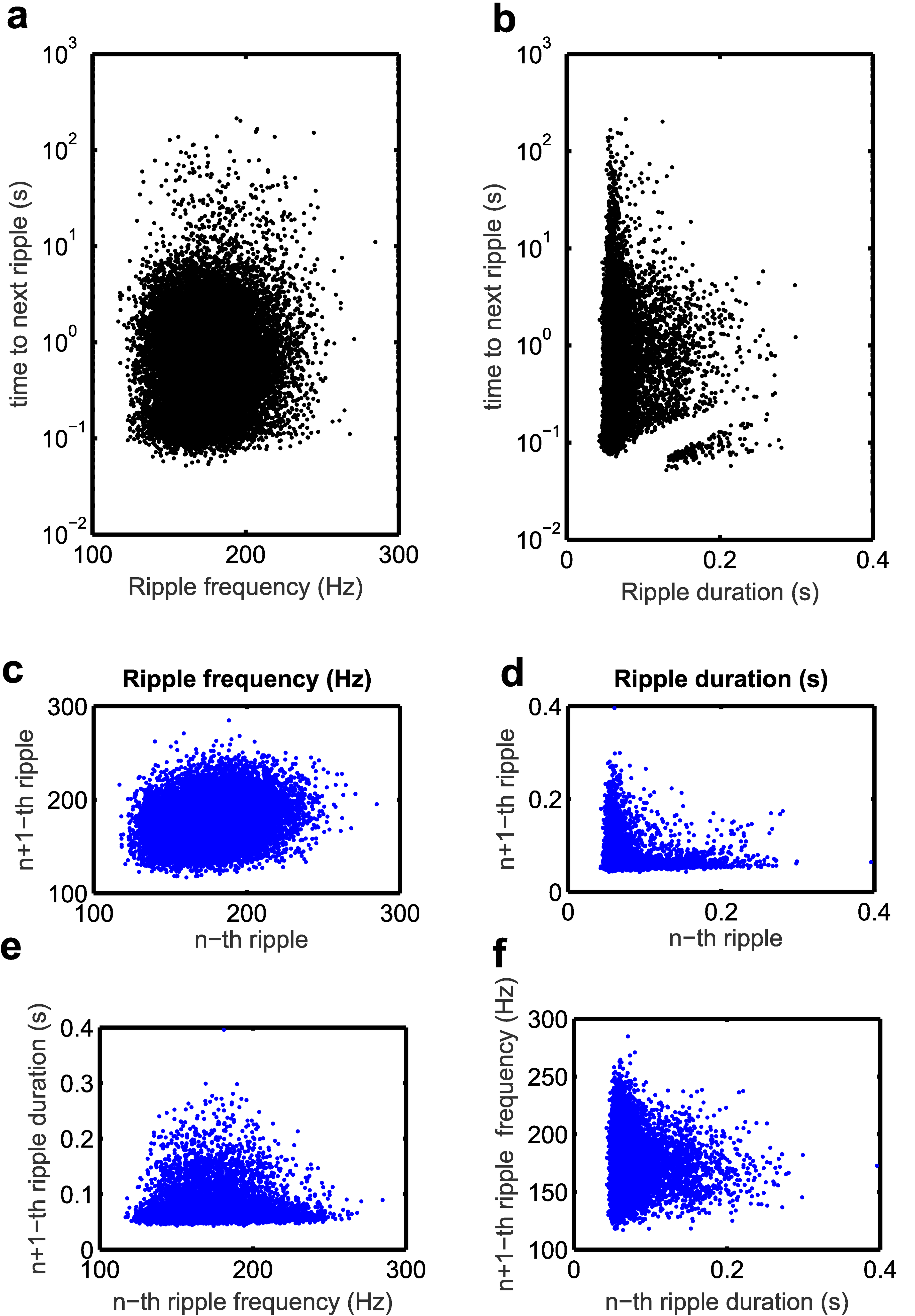

Supplement: S2 Fig — For each ripple in a 25hr in vivo experiment, its frequency is plotted against the time to the next ripple (a). Its duration is plotted against the time to the next ripple (b). Note that there is no clear correlation between the time to the next ripple and current ripple frequency or duration. Poincare return maps relate a property of the current ripple (n-th ripple) to the same property (or a different one) in the next ripple (n+1-th ripple). If the system has memory, then a property of a ripple is predictive of the same (or a different) property in the next ripple. In a system with no memory, the cloud distribution should look like the direct product of the distributions of the two properties considered. (c) Ripple frequency does not show an obvious memory effect. Note that the distribution in n vs n+1 looks like the direct product of Fig 1B with itself. (d) Ripple duration does not show memory effect across ripples. Compare with Fig 1D times (outer product) itself. (e) Current ripple frequency does not influence next ripple duration. (f) Current ripple duration does not affect next ripple frequency. (TIF) [file pcbi.1004880.s002.tif]

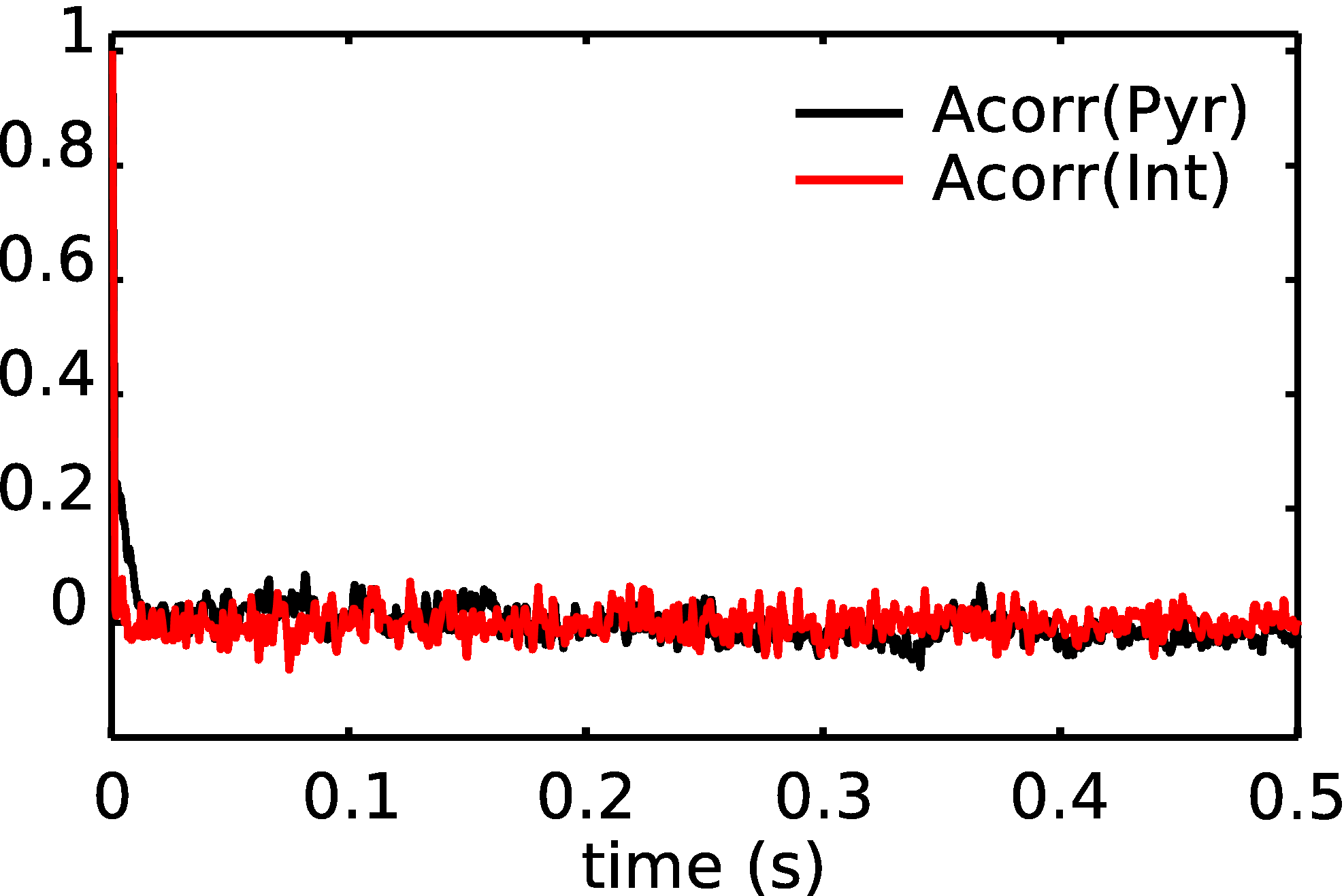

Supplement: S3 Fig — (TIF) [file pcbi.1004880.s003.tif]

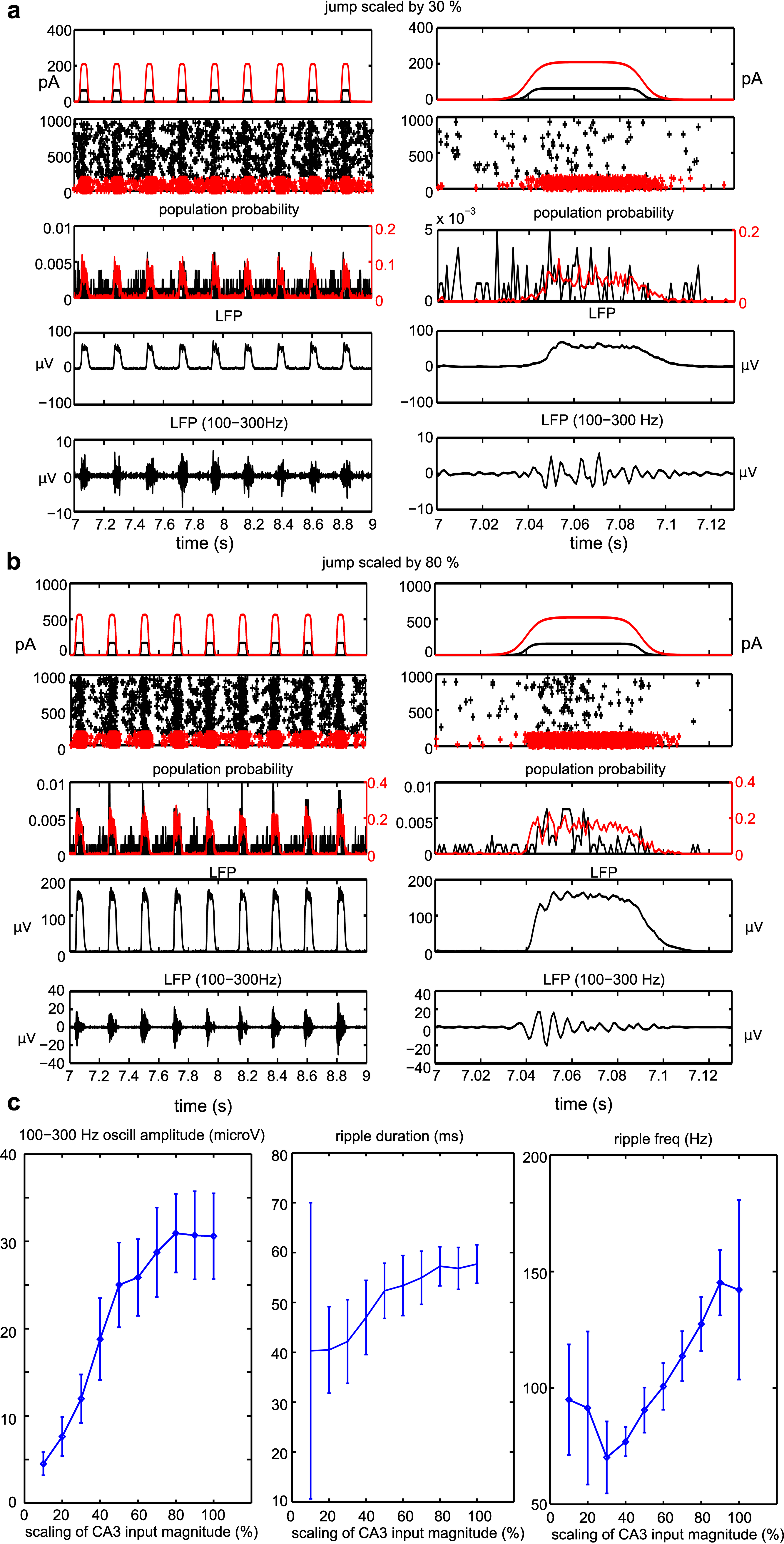

Supplement: S4 Fig — (a) Example of a simulation in which CA3-mediated input current in both pyramidal cells and interneurons of the CA1 model is reduced to 30% of its baseline magnitude, by multiplying Iinp(t) by 0.3. Note the size of y-axis on the top panels. The right column shows a smaller time interval, so that the “ripple” profile can be seen. Time is in seconds in all panels. Top panels: current input (in pA) from CA3 to pyramidal cells (black) and interneurons (red). Second panels from the top: rastergram of pyramidal cells (black) and interneuron (red) spikes. Middle panels: probability of spiking for pyramidal cells (black) and interneuron (red) populations, in 1ms time bins. Last two panels: wide band (above) and filtered (100–300 Hz) LFP trace (in μV). (b) Same as in (a), but for CA3-mediated current only scaled to 80% of its baseline strength. Note how the interneuron population fires more organized, which results in a filtered LFP more structured in this case, compared to the 30% scaling. (c) Summary plot of core ripple properties when the input from CA3 to both pyramidal cells and interneurons is scaled in a range of 10–100%. Ripple amplitude rises from 5 μV (undetectable) as input size increases, and saturates between 80–100% of the input. Ripple duration is ill-defined at 10% input (note the great variability as a number of events that qualify for ripple detection do not show enough oscillations for the duration to be consistently estimated), and increases with increasing input amplitude. Ripple frequency is over-estimated below 30% due to the 100–300 Hz filtering in ripple detection, but once input is above 30% one can see the shift from high-gamma to ripple range, controlled by input size. (TIF) [file pcbi.1004880.s004.tif]

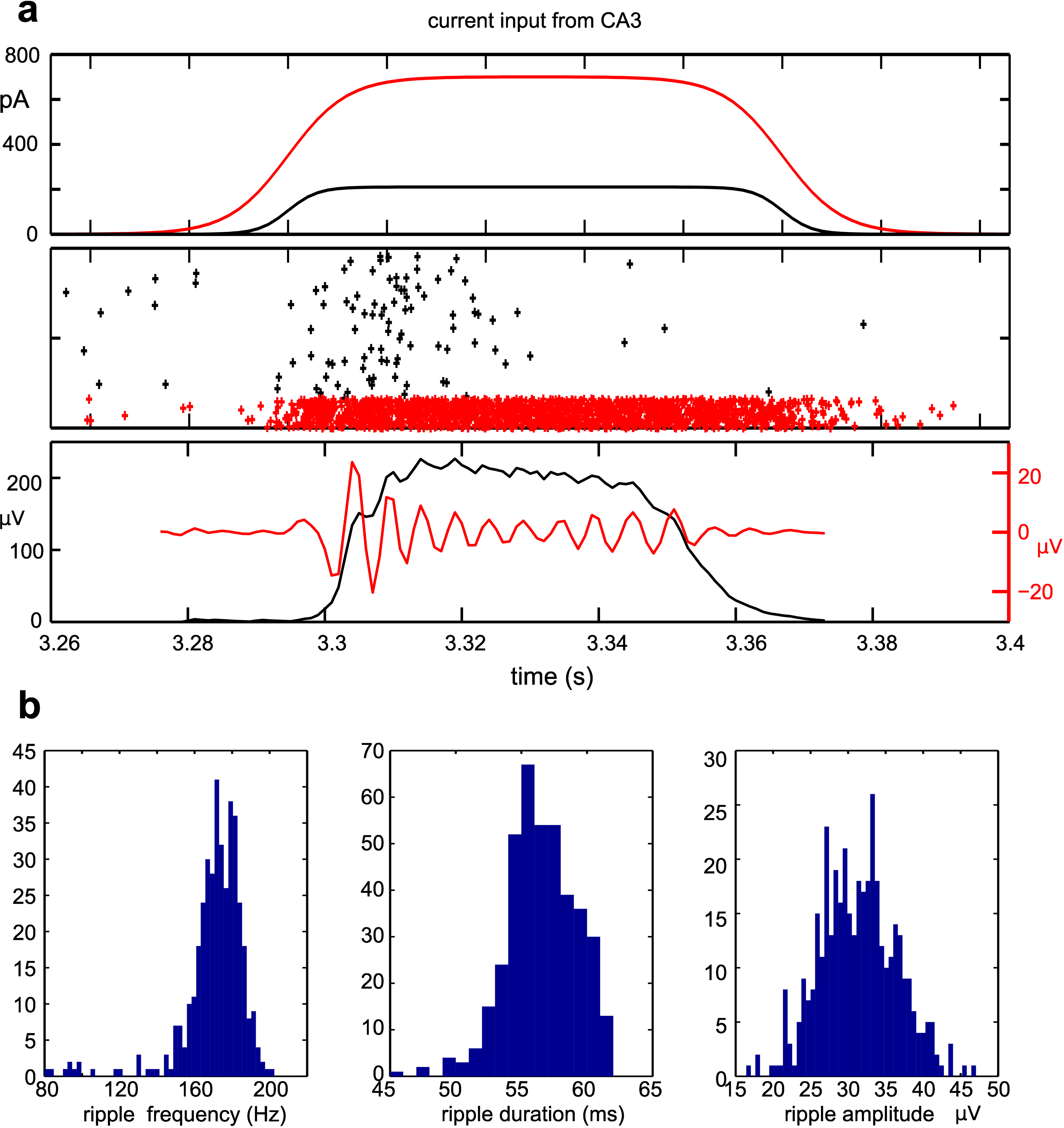

Supplement: S5 Fig — (a) Example of a typical ripple event in the network when I-to-I synapses are removed. Top: input current (in pA) from CA3 to pyramidal cells (black) and interneuron (red) population. Middle: rastergram of pyramidal cells (black) and interneurons (red) spikes during a ripple. Lower plot: wide-band (black) and filtered (100–300 Hz, red) LFPs in the network. Note that the oscillations stop much quicker than in the network with I-to-I inhibition shown in Fig 2. (b) Summary histograms for ripple frequency (Hz), duration (ms) and amplitude (μV) in the case of removed I-to-I synapses. The overall properties of ripples are on average preserved (as expected), yet the filtered LFP is unable to ever generate ripples longer than 60m, compare with Fig 3B. (TIF) [file pcbi.1004880.s005.tif]
